# Glucagon, cyclic AMP, and hepatic glucose mobilization: A half‐century of uncertainty

**DOI:** 10.14814/phy2.15263

**Published:** 2022-05-15

**Authors:** Robert L. Rodgers

**Affiliations:** ^1^ Department of Biomedical and Pharmaceutical Sciences College of Pharmacy University of Rhode Island Kingston Rhode Island USA

## Abstract

For at least 50 years, the prevailing view has been that the adenylate cyclase (AC)/cyclic AMP (cAMP)/protein kinase A pathway is the predominant signal mediating the hepatic glucose‐mobilizing actions of glucagon. A wealth of evidence, however, supports the alternative, that the operative signal most of the time is the phospholipase C (PLC)/inositol‐phosphate (IP3)/calcium/calmodulin pathway. The evidence can be summarized as follows: (1) The consensus threshold glucagon concentration for activating AC ex vivo is 100 pM, but the statistical hepatic portal plasma glucagon concentration range, measured by RIA, is between 28 and 60 pM; (2) Within that physiological concentration range, glucagon stimulates the PLC/IP3 pathway and robustly increases glucose output without affecting the AC/cAMP pathway; (3) Activation of a latent, amplified AC/cAMP pathway at concentrations below 60 pM is very unlikely; and (4) Activation of the PLC/IP3 pathway at physiological concentrations produces intracellular effects that are similar to those produced by activation of the AC/cAMP pathway at concentrations above 100 pM, including elevated intracellular calcium and altered activities and expressions of key enzymes involved in glycogenolysis, gluconeogenesis, and glycogen synthesis. Under metabolically stressful conditions, as in the early neonate or exercising adult, plasma glucagon concentrations often exceed 100 pM, recruiting the AC/cAMP pathway and enhancing the activation of PLC/IP3 pathway to boost glucose output, adaptively meeting the elevated systemic glucose demand. Whether the AC/cAMP pathway is consistently activated in starvation or diabetes is not clear. Because the importance of glucagon in the pathogenesis of diabetes is becoming increasingly evident, it is even more urgent now to resolve lingering uncertainties and definitively establish glucagon’s true mechanism of glycemia regulation in health and disease.

## Graphical abstract

The prevailing view is that glucagon promotes hepatic glucose mobilization by activating adenylate cyclase and generating the intracellular signal cyclic AMP. This review summarizes evidence supporting the alternative view that the true intracellular signal mediating glucagon’s action is instead inositol‐triphosphate (IP3) pathway.

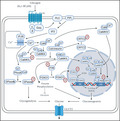

## 1 INTRODUCTION

The metabolic hormone glucagon stimulates the movement of glucose from the liver into the bloodstream. Its main partner, insulin, has a complementary role, to oppose the hepatic glucose‐mobilizing effects of glucagon while promoting the movement of glucose out of the bloodstream into glucose‐utilizing tissues such as muscle and adipose. When in balance, the two hormones effectively maintain euglycemia under variable nutritional and metabolic conditions (Campbell & Newgard, 2021; Zhang et al., 2019).

The central thesis of this review is that glucagon only activates a “basic” cellular signal in liver most of the time, under normal conditions of physical activity and systemic glucose demand. During episodes of elevated glucose demand, as in the early postnatal period or in the exercising adult, glucagon activates both the basic and a “backup” signal simultaneously, boosting the hepatic contribution to systemic glucose supply. Glucagon activates the backup signal much less consistently in the metabolically stressful conditions of starvation and diabetes. The backup signal was characterized first, in 1971, which helps to explain why it has since been the more extensively studied. Even now, it is regarded by most investigators as the main or even exclusive cellular signal pathway mediating the hepatic glucose‐mobilizing effects of glucagon most or all of the time. The discovery of the basic pathway was not published until fifteen years later, but only more recently has it begun to receive the attention that it deserves.

The current model of glucagon’s hepatic glucose‐mobilizing actions had its origins in the work of the Nobel laureate Earl Sutherland and his colleagues in the early 1970s.

## 2 DISCOVERY OF THE BACKUP SIGNAL

In 1971, the year that he was awarded the Nobel Prize for his discovery and chemical characterization of the first known cellular signal (“second messenger”) molecule, cyclic adenosine‐3’,5’‐ monophosphate (cAMP) (Sutherland, 1972), Earl Sutherland—along with his colleagues John Exton, Al Robison, and Charles Park ‐ published a groundbreaking paper on metabolic effects of glucagon on the liver (Exton, Lewis, et al., 1971). They used glucagon, epinephrine, and norepinephrine as probes to stimulate the production of cAMP and the mobilization of glucose in the isolated perfused rat liver. They calculated that the threshold concentration (TC) of glucagon required to generate measurable levels of cAMP in the tissue was 2 × 10^−10^ M (200 pM). Since then, the majority of studies, largely on perfused livers, hepatocytes, or hepatocyte membranes (see below), collectively indicate that the TC is half that, at or near 1 × 10^−10^ M (100 pM).

Sutherland and coworkers proposed that concentrations of glucagon in the plasma perfusing the liver need to reach that threshold in order to activate the signal in vivo: “It is important to consider whether the concentrations of glucagon … promoting cyclic AMP accumulation in the isolated liver, lie within the normal range in portal venous blood.” To provide supporting evidence that they do, the authors cited two reports that had been published two years earlier (Buchanan et al., 1969; Ohneda et al., 1969). According to those studies, plasma glucagon levels in the canine pancreatico‐duodenal vein ranged between around 200 and 1000 pM. On that basis, they concluded that “…the minimal effective concentration of glucagon for the promotion of hepatic cyclic AMP accumulation and glucose mobilization observed in the present study (2 × 10^−10^ M) would not be out of line with the probable level of glucagon in portal venous blood”. That statement, possibly more than any other, has directed the focus on cAMP as the main or exclusive intracellular mediator of glucagon’s actions ever since. In retrospect, however, the plasma glucagon estimates in the reports that they had cited appear to have been erroneously high. A wealth of subsequent studies confirms that glucagon concentrations in the hepatic portal and extrahepatic circulations of adult mammals, under normal conditions, are well below the 100 pM TC required to activate adenylate cyclase (AC), enhance the production of cAMP, and activate protein kinase A (PKA) in perfused livers, hepatocytes, and hepatocyte membranes (Rodgers, 2012).

A fundamental and persistent problem with the current model, rarely acknowledged or addressed, is that there is not enough glucagon in the hepatic portal circulation to activate AC in liver most of the time.

## 3 PLASMA GLUCAGON CONCENTRATIONS

**FIGURE 1:**
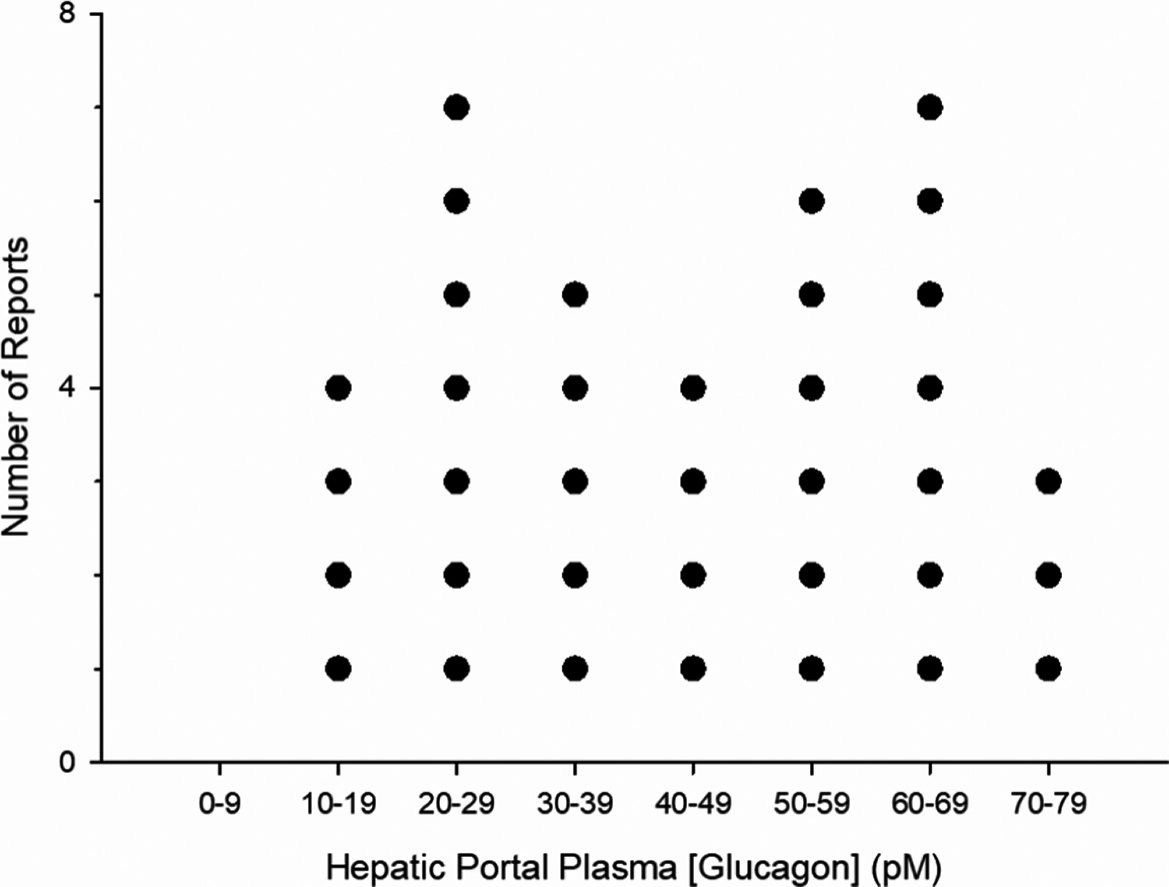
Distribution of hepatic portal plasma glucagon concentrations (pM) in fed or fasted humans, dogs, rats, and miniature pigs, measured by radioimmunoassay (RIA). Durations of fasting or starvation were 12–48 h. The 36 values are means determined by RIA between 1976 and 2017. Plasma samples were obtained from fed, fasted, starved, conscious, or anesthetized animals. Twenty of the 36 mean values were obtained using “Unger’s 30K antibody”, with most of the remainder using commercial RIA kits. Statistical values: Composite mean = 43.9 pM; SD = 19.8; SEM =3.3; and the 99.9999% confidence interval =16.1, for a statistical range of 27.8 – 60.0 pM. There were no obvious correlations between plasma concentration and species, nutritional state, or RIA method. Note that all 36 values are below the consensus TC of 100 pM for activating AC (see Figure 4). The 36 sources were: Kraft et al. (2017), Lewis et al. (1997), Saccà et al., (1979) and Vaitkus et al., (1984), (10–19); Berger et al. (1994), Blommaart et al. (1995), Curnow et al. (1976), Fries et al. (1982), Hickman et al. (1992), Imai et al. (2003) and Wasserman et al. (1993) (20–29); Androgué et al. (1985), Baumann et al. (1981), Cersosimo et al. (1989), Rao (1995) and Sherwin et al. (1977) (30–39); Francavilla et al. (1980), Holst et al. (1980), Langhans et al. (1984) and Müller et al. (1984) (40–49); Demigné et al. (1985), Goldstein and Curnow (1978), Horikawa et al. (1998), Jaspan et al. (1984), Silva et al. (1990) and Wolf and Eisenstein (1981), (50–59); Balks and Jungermann (1984), Gannon and Nuttall (1993), Ishida et al. (1981), Kinoshita et al. (1985), McLeod et al. (1983), Rabouti et al. (1989) and Silva et al. (1990) (60–69); Hussein et al. (1986), Latour et al. (1999) and Okuda et al. (1994) (70–79)

**FIGURE 2:**
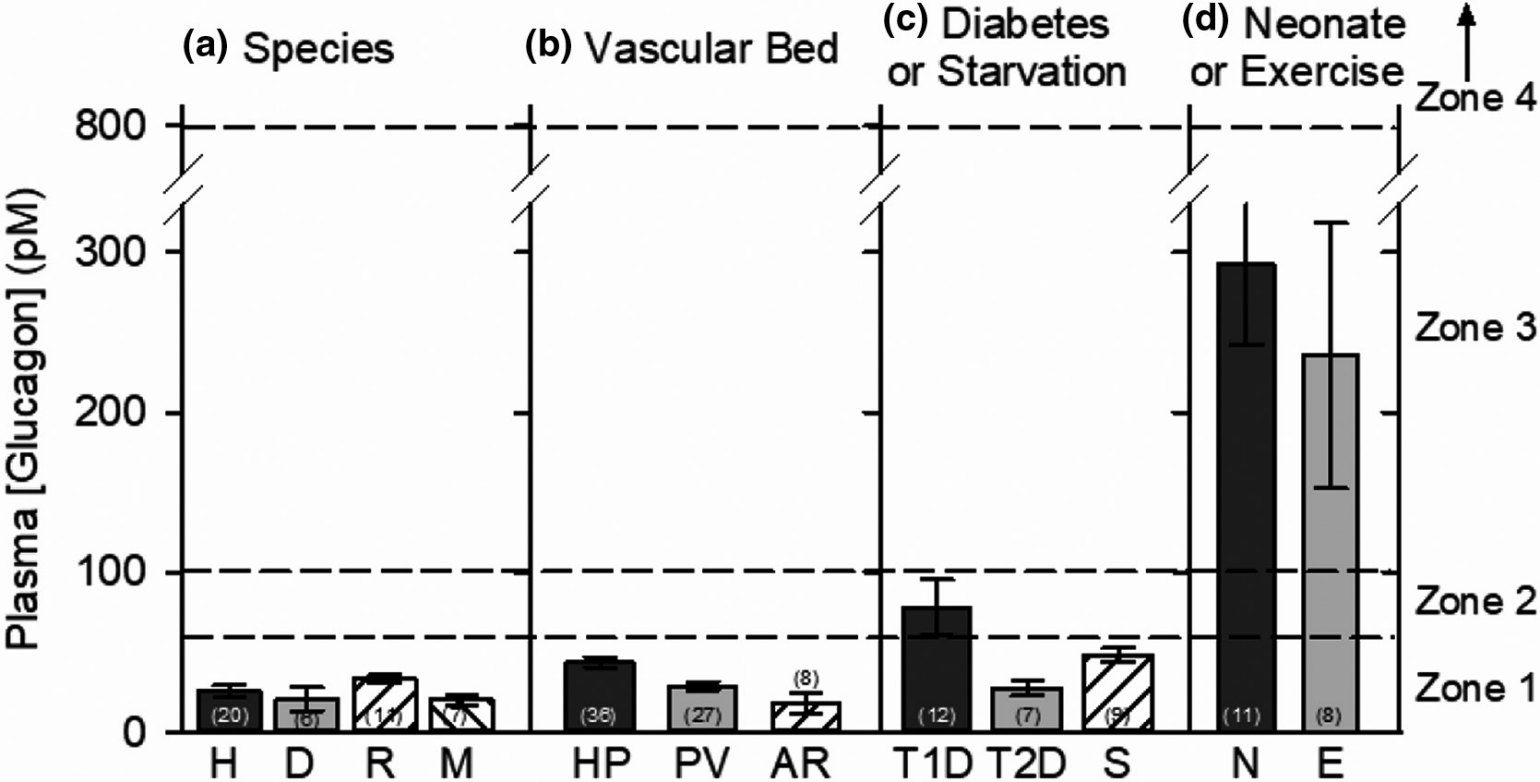
Plasma glucagon concentrations as measured by radioimmunoassay (RIA). Mean values ±SEM are grouped by species (a), vascular bed (b), and conditions (c and d), gathered from a large sampling published between 1969 and 2020. (a) Mean peripheral venous plasma concentrations in fed and short‐term fasting (less than 24 h) adult humans (H), dogs (D), rats (R), and mice (M). When fasted, the durations were 8–12 hours in humans, 13–24 h in dogs, 12–16 h in rats, and 14–16 h in mice. (b) Plasma levels in hepatic portal (HP; see Figure 1), peripheral venous (PV), and arterial (AR) circulations. The mean values in the peripheral venous and arterial circulations are 67% and 32%, respectively, of those in the hepatic portal plasma. (c) Mean peripheral venous plasma glucagon levels in type 1 diabetes (T1D; STZ‐ or alloxan‐induced diabetes in mice and rats), type 2 diabetes or obesity with insulin resistance in humans (T2D), and in starvation (S) of more than 24 h. Durations of starvation were 2.25–6 days in rats and 3–7 days in humans. (d) Mean peripheral venous plasma glucagon levels in neonates (N) and exercising adults (E). The ages of neonates ranged between newborn and 4 days in humans (*n* = 5) and between newborn and 14 days in mice and rats (*n* = 8). Durations of exercise in rats, dogs, and humans varied from 1 h to exhaustion. The numbers in parentheses indicate the number of publications from which the data were averaged. Data from some references applied to more than one grouping. Plasma concentrations are divided here into four zones based on RIA estimates: Zone 1 (normal physiological) between 0 and 60 pM, spanning the statistical range of mean glucagon concentrations of normal, unstressed adult mammals (Figure 1); Zone 2 (transitional) between 60 and 100 pM, the upper limit of which is the consensus TC for activating AC in dose‐response curves generated ex vivo (see text and Figure 4); Zone 3 (physiological hyperglucagonemia) between 100 and 800 pM, includes the highest mean plasma glucagon concentration in neonates (600 pM) (Blommaart et al., 1995) or exercising adults (732 pM) (Seitz et al., 1999) among the references cited here; and Zone 4 (pharmacological) between 800 and 1,000,000 pM, the highest glucagon concentration reported to maximally activate AC in dose‐response curves generated ex vivo (see text and Figure 4). One value in B (PV) was obtained by ELISA instead of RIA (Hussein et al., 1986). The data were compiled from the following references: A (Species)–Humans (H): Bolli et al. (1984), Borghi et al. (1984), Brodows (1985), Evans et al. (2004), Fujita et al. (1975), Gosmanov et al. (2005), Hamaguchi et al. (1991), Hansen et al. (1982), Heise et al. (2004), (2004), Henkel et al. (2005), Jaspan et al. (1984), Kalkhoff et al. (1973), Livingston et al. (1985), Okba et al. (2020), Petersen and Sullivan (2001), Porcellati et al. (2003), Raju and Cryer (2005), Sherwin et al. (2005), Tasaka et al. (1980), Verillo et al. (1988); Dogs (D): Cersosimo et al.(1998), Coker, Koyama, Brooks, et al. (1999), Kraft et al. (2017), Ishida et al. (1983), Moore et al. (2014), Sherck et al. (2001), Sindelar et al. (1998), and Vaitkus et al. (1984); Rats (R): Balks and Jungermann (1984), Charbonneau et al. (2005), Langhans et al. (1984), Latour et al. (1999), Mayor and Calle, (1988), Omer et al. (2004), Powell et al. (1993), Ruiter et al. (2003), Unger (1985), Widmaier et al. (1991) and Winzell et al. (2007), Mice (M): Green et al. (2016), Karlsson et al. (2002), Marty et al. (2005), Parker et al. (2002), Perry et al. (2020), Winzell et al. (2007) and Zhang et al. (2018) B (Vascular Bed) ‐ Hepatic Portal (HP): See legend, Figure 1, Peripheral Venous (PV): Balks and Jungermann (1984), Bolli et al. (1984), Cersosimo et al. (1998), Charbonneau et al. (2005), Coker, Koyama, Brooks, et al. (1999), Hamaguchi et al. (1991), Hansen et al. (1982), Heise et al. (2004), Henkel et al. (2005), Ichikawa et al. (2019), Ishida et al. (1981), Jaspan et al. (1984), Karlsson et al. (2002), Langhans et al. (1984), Latour et al. (1999), Livingston et al. (1985), Marty et al. (2005), Mayor and Calle (1988), Nair et al. (1987), Omer et al. (2004), Parker et al. (2002), Perry et al. (2020), Powell et al. (1993), Raju and Cryer (2005), Shi et al. (1996), Wall et al. (2005) and Winder, Arogyasami, et al. (1988), Arterial (AR): Balks and Jungermann, (1984), Carlson and Winder (1999), Coker, Koyama, Brooks, et al. (1999), Jackson et al. (2004), Moore et al. (2014), Patel (1984), Pencek et al. (2004) and Sherck et al. (2001), C (Diabetes or Starvation) ‐Type 1 Diabetes (T1D): Chamras et al. (1980), Green et al. (2016), Hermida et al. (1994), Mayor and Calle (1988), Meek et al. (2015), Patel (1984), Shi et al. (1996), Srikant et al. (1977), Walsh and Dunbar (1984), Widmaier et al. (1991), Yamashita et al. (1980) and Zhang et al. (2018), Type 2 Diabetes (T2D): Bolli et al. (1984), Borghi et al. (1984), Hamaguchi et al. (1991), Henkel et al. (2005), Knop et al. (2005), Marliss et al. (1970), Nair et al. (1970); Starvation: Aguilar‐Parada et al. (1969), Bois‐Joyeux et al. (1986), Brodows (1985), Goldstein et al. (1978b), Hamaguchi et al. (1991), Henkel et al. (2005), Knop et al. (2007), Marliss et al. (1970), Mlekusch et al. (1981), Nair et al. (1987), Seitz et al. (1976), Smadja et al. (1990), Srikant et al. (1977), Verrillo et al. (1988), D (Neonates or Exercising Adults) ‐ Neonates (N): Blommaart et al. (1995), Fernández‐Milán et al. (2013), Girard et al. (1973), Luyckx et al. (1972), Lyonnet et al. (1988), Milner et al. (1972), Movassat et al. (1997), Nurjhan et al. (1985), Ogata et al. (1988), Salle and Ruiton‐Ugliengo (1977) and Sperling et al. (1974), Exercise (E): Carlson and Winder (1999), Charbonneau et al. (2005), Coker, Koyama, Lacy, et al. (1999), Latour et al. (1999), Sellers et al. (1988), Winder, Arogyasami, et al. (1988), Winder et al. (1981) and Winder, Yang, et al. (1988)

**FIGURE 3:**
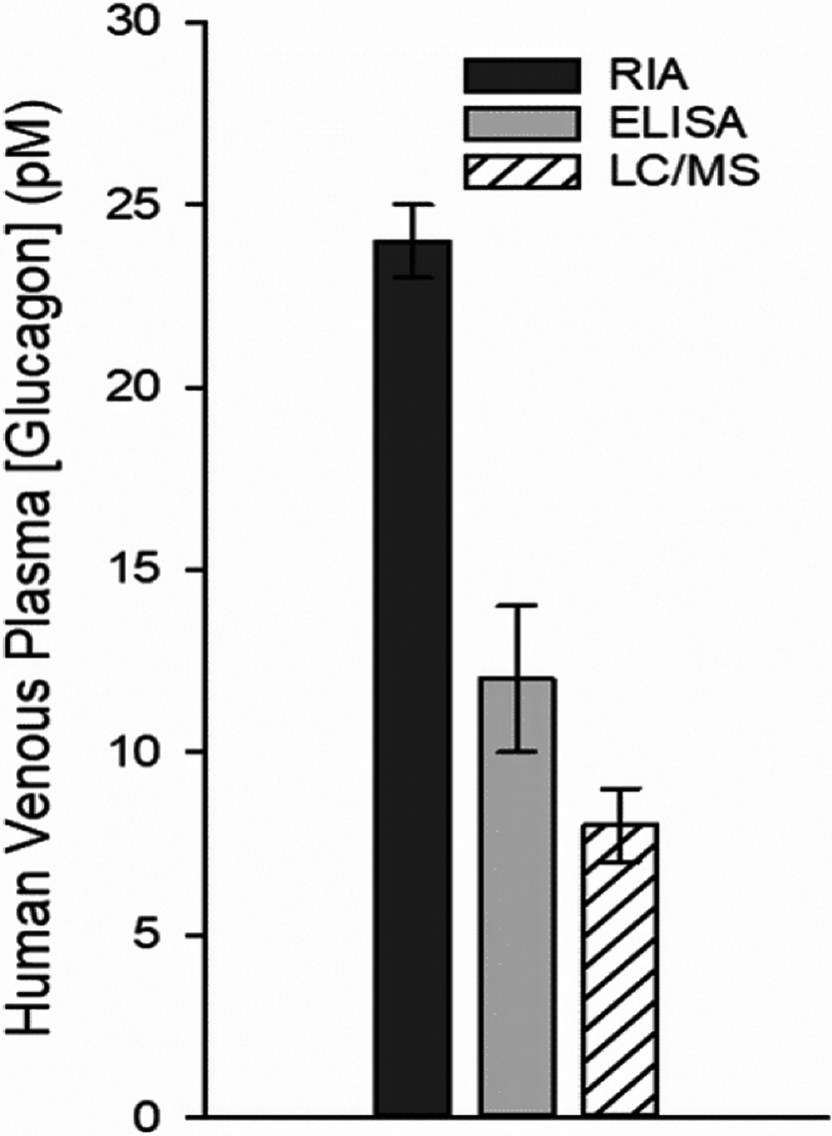
Comparison of plasma glucagon concentrations measured by RIA, double‐antibody sandwich enzyme‐linked immunosorbent assay (ELISA), and liquid chromatography/mass spectrometry (LC/MS). Values were obtained from peripheral venous blood samples taken from 13 healthy human volunteers. The authors included sitagliptin, an inhibitor of dipeptidyl peptidase‐4, in their assay solution, a precaution not always taken. They did not specify whether they assayed fresh or frozen‐thawed samples. Notably, the mean values as assessed by ELISA and LC/MS were 50% and 33%, respectively, of those measured by RIA. The figure is adapted from Miyachi et al. (2017). The data clearly reveal the profound influence of assay technique on estimates of plasma glucagon concentrations

In the two reports of hepatic portal plasma glucagon concentrations cited by Sutherland and colleagues, the authors used radioimmunoassay (RIA) techniques to arrive at their estimates. But RIA‐based methods, especially those that were applied in the 1960’s, were generally not optimal in terms of accuracy, specificity, and sensitivity (Aguilar‐Parada et al., 1969; Bak et al., 2014; Wewer Albrechtsen et al., 2014; Wewer Albrechtsen, Kuhre, Pedersen, et al., 2016; Wewer Albrechtsen, Kuhre, Windeløv, et al., 2016; Wewer Albrechtsen, Veedfald, et al., 2016). Peptides whose sequences partially overlap that of glucagon, including glicentin and oxyntomodulin, can interfere, producing erroneously high readings (Bak et al., 2014; Holst, 1983, 2014). Further, glucagon is subject to degradation in plasma or when stored in the freezer for long periods (Deacon et al., 2003; Wewer Albrechtsen et al., 2015) or when incubated with liver membranes (Baumann et al., 1981), and recovery is not consistently reported. Estimates can also differ depending on the part of the glucagon molecule for which the antibody has affinity (Deacon et al., 2003; Soybel et al., 1983; Trebbien et al., 2004). For example, RIA antibodies directed at the carboxyl, amino, and mid‐region of the molecule yielded values of 16, 34, and 58 pM, respectively, in the same plasma sample (Deacon et al., 2003). Largely because they may not rely on the same antibody, various commercial RIA kits can yield estimates that are different from each other and from those produced by in‐house RIAs (Bak et al., 2014; Wewer Albrechtsen et al., 2014). As an acknowledgement of uncertainties implicit in the concentration estimates obtained by RIA, the hormone is commonly designated “immunoreactive glucagon”. In recent years, enzyme‐linked immunosorbent assays (ELISA) have been gradually replacing standard RIA techniques as the method of choice because they have been demonstrated to be, on the whole, more specific and sensitive, and thus more accurate, than conventional RIAs are (Holst, 1983, 2014). Predictably, they also generally yield lower values than RIA techniques do (Ichikawa et al., 2019; Miyachi et al., 2017). Nevertheless, until recently, by far the largest portion of the reported measurements of plasma glucagon levels over the last half‐century have been determined by RIA. In the following sections, plasma glucagon concentrations can be assumed to be those in the peripheral venous circulation determined by RIA unless otherwise specified (Figures 1, 2, 3, Table 1).

**TABLE 1:** Comparisons of plasma glucagon concentrations as measured by ELISA and RIA

Condition	Plasma glucagon concentration (pM)			
	ELISA	RIA	ELISA/RIA	Reference
Normal	6	22	0.27	Ichikawa et al. (2019)
Prediabetic	10	23	0.43	Ichikawa et al. (2019)
Diabetic	13	27	0.48	Ichikawa et al. (2019)
Normal	12	24	0.50	Miyachi et al. (2017)
Obese, NGT	13	27	0.48	Wewer Albrechtsen et al. (2014)
Mean ±SEM	10.8 ± 1.3	24.6 ± 1.0	0.43 ± 0.04	

Note

**FIGURE 4:**
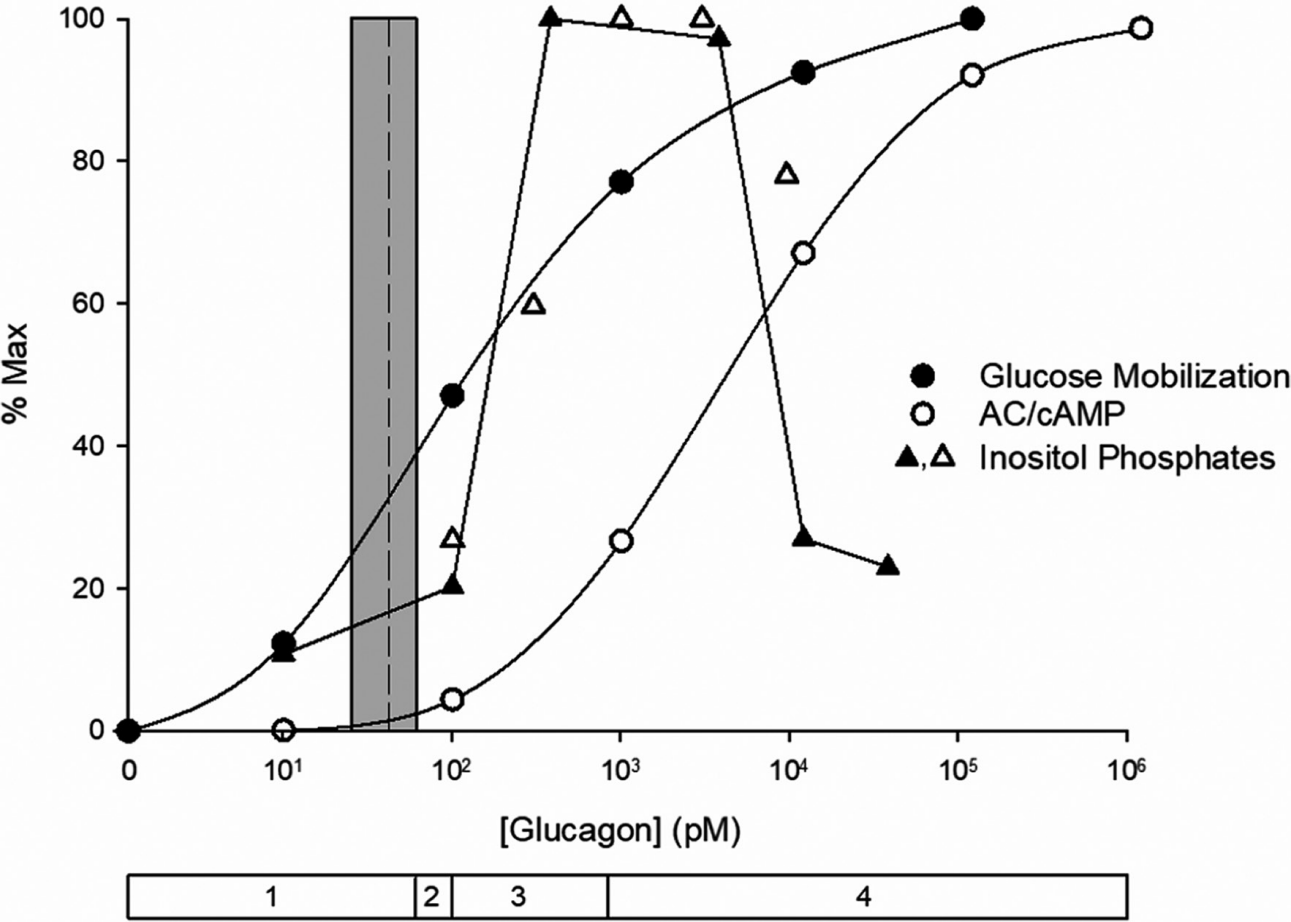
Glucagon concentrations required to stimulate glucose mobilization compared to those required to activate the PLC/IP3 and AC/cAMP pathways. The shaded vertical bar is the statistical range of mean glucagon concentrations, determined by RIA, in the hepatic portal system of humans, rats, dogs, and pigs, 27.8–60.0 (10^1.44^–10^1.78^) pM, with a mean (dotted line) of 43.9 pM (10^1.64^) pM (see Figure 1). The glucose mobilization curve (glucose output, glycogenolysis, or gluconeogenesis) is a composite of 11 dose‐response curves published between 1971 and 1999 (Bizeau and Hazel (1999), Cárdenas‐Tanús et al. (1982), Chan et al. (1979), Corvera and García‐Sáinz (1984), Exton, Lewis, et al. (1971), Felíu et al. (1976), Fleig et al. (1984), Hermsdorf et al. (1989), Ikezawa et al. (1998), Khan et al. (1980) and Wernette Hammond and Lardy (1985)), determined in rat hepatocytes or isolated perfused rat livers. The composite AC/cAMP curve is the average of 15 individual curves generated in rat hepatocytes, liver membranes, or cell lysates (Clark and Jarrett (1978), Corvera and García‐Sáinz (1984), Dich and Gluud (1976), Dighe et al. (1984), England et al. (1983), Exton, Robison, et al. (1971), Hermsdorf et al. (1989), Lynch et al. (1989), Pohl et al. (1972), Robberecht et al. (1988), Rodbell et al. (1971), Soman and Felig (1978), Sonne et al. (1978), Unson et al. (1989) and Yagami (1995)). The inositol‐phosphate data are from Wakelam et al. (1986) (filled triangles) and Unson et al. (1989) (open triangles). The bar below the X axis indicates the four concentration zones as depicted in Figure 2. Glucagon clearly activates the PLC/IP3 pathway in zones 1–4. However, in vivo it does not activate AC in zone 1, inconsistently activates it in zone 2 (see text), and predictably activates AC in zones 3 and 4. The TC for activating AC ex vivo,100 pM, is 2.5 times greater than the aggregate mean hepatic portal plasma concentration of 44 pM, and 100‐fold greater than the 10 pM TC for increasing glucose mobilization. The data clearly show that a substantial fraction of the maximum stimulation of hepatic glucose mobilization, around 40%, is produced by glucagon at physiological (zone 1) concentrations and mediated exclusively by the PLC/IP3 pathway. An additional 35%, generated by supraphysiological, zone 3 concentrations, is mediated by maximal activation of the PLC/IP3 pathway together with a submaximal activation AC/cAMP pathway. The resulting boost in glucose output within zone 3 may be an adaptive response to elevated systemic glucose demand in vivo (see text)

Values are from peripheral venous samples taken from nondiabetic or T2D humans. The mean value as measured by RIA in this group is close to the collective estimates obtained from nondiabetic and T2D humans in Figure 1 A and C: 26 and 27 pM, respectively. According to the ELISA/RIA ratio calculated here of 0.43, the mean concentration in the hepatic portal circulation in fed or short‐term fasting nondiabetic adults, as measured by RIA, of 44 pM (Figure 1) would be at or near 19 pM if measured by ELISA, 1/5 of the TC for AC activation, 100 pM, determined ex vivo (Figure 4). NGT, non‐glucose‐tolerant.

As Sutherland and coworkers proposed, the validity of the cAMP hypothesis – that the AC/cAMP pathway mediates the hepatic glucoregulatory actions of glucagon – is critically dependent on establishing that hepatic portal plasma concentrations of glucagon are sufficient to activate AC in liver. A large body of evidence gathered since then confirms that most of the time they are not. According to 36 reports published between 1976 and 2017, the composite mean hepatic portal plasma glucagon concentration, measured by RIA, in metabolically unstressed, fed or fasted humans, dogs, rats, and pigs at rest, conscious or under anesthesia, is 43.9 ± 3.3 pM (Figure 1), with a 99.9999% confidence interval of 27.8–60.0 pM. Note that all of the individual mean values shown in Figure 1 are below 100 pM, the consensus TC for activating AC ex vivo (see below). According to these results, there is not nearly enough glucagon in hepatic portal plasma—in either anesthetized or conscious animals at rest—to activate AC in liver most of the time. But they can occasionally rise to levels sufficient to activate AC during intervals of elevated metabolic stress (discussed below).

For purposes of this review, plasma glucagon concentrations are divided into four zones (Figure 2a; Coker, Koyama, Brooks, et al., 1999). Categorizing them this way highlights the importance of distinct plasma concentration ranges as they apply to the hormone’s mechanism of action under varying conditions. Zone 1 is between 0 and 60 pM, the statistical hepatic portal glucagon concentration range under normal conditions as described above and in the legend for Figure 1. It can be defined as “physiological glucagonemia,” spanning plasma glucagon concentrations in the largest group, fed or fasting (less than 24 h and usually overnight) healthy adult experimental animals and humans at rest or during normal physical activity. Mean concentrations reported in the literature for this group consistently fall well within zone 1 regardless of species (human, dog, rat, or mouse; Figure 2a) or vascular bed (hepatic portal, peripheral venous, or arterial) (Figure 2b). Mean plasma glucagon concentrations of rabbits, pigs, cats, and cattle are also within zone 1 (20–50 pM) (Brand et al., 1996; Deacon et al., 2003; Kavianipour et al., 2003; Trebbien et al., 2004; Uvnäs‐Moberg et al., 1986; Williams et al., 2006). Of course, concentrations in the hepatic portal circulation are higher than those in peripheral vascular beds because the former is between the pancreas and the liver. Note here that, according to the reports cited in Figure 2, mean plasma glucagon concentrations in the hepatic portal circulation are 50% and 140% higher than they are in the peripheral venous and arterial vascular beds respectively (Figure 2b), reflecting the hepatic extraction and distribution of the hormone (Balks & Jungermann, 1984; Dobbins et al., 1995; Ishida et al., 1981; Jaspan et al., 1984; Trebbien et al., 2004). As discussed in more detail below, when circulating glucagon concentrations vary within zone 1, as they do in the metabolically unchallenged mammal at rest (Balks & Jungermann, 1984), hepatic AC activity and tissue cAMP levels are constitutive (basal), and not affected by the hormone.

Zone 2 is between 60 and 100 pM. The upper limit of zone 2 is the consensus TC for AC activation determined ex vivo. The zone 2 concentration range is described here as “transitional” because glucagon at concentrations within that range can stimulate hepatic AC activity, but inconsistently and unpredictably. Mean plasma glucagon concentrations in experimental insulin‐dependent diabetes (T1D) or starvation can be in zone 1 or zone 2 (Figure 2c), with variable effects on AC activity. Mean plasma concentrations in human type 2 diabetics (T2D), by contrast, are uniformly within zone 1 (Figure 2c), and therefore would not be expected to activate AC in that condition.

Zone 3, which is categorized here as “physiological hyperglucagonemia,” ranges between 100 and 800 pM. Two groups whose mean plasma glucagon concentrations usually fall into this range are early neonates and exercising adults (Figure 2d). The upper limit of 800 pM was selected to capture the full range of mean plasma concentrations in those two groups. Among the sources cited here (the citation list is in the legend of Figure 2), the highest reported mean plasma concentration in early neonates was 600 pM (Blommaart et al., 1995), and in exercising adults was 732 pM (Sellers et al., 1988). When plasma glucagon concentrations enter zone 3, they predictably activate the AC/cAMP pathway.

Zone 4, from 800 pM to as high as 1,000,000 pM, can be described as “pharmacological,” achieved in vivo only under extraordinary circumstances or produced by administration of high concentrations ex vivo or high doses of exogenous glucagon in vivo. For example, peak mean plasma concentrations after a dose of 1 mg, administered to severely hypoglycemic diabetic patients, can reach 1,300 pM (Blauw et al., 2015). Examples of conditions or experimental manipulations that result in zone 3 or zone 4 plasma concentrations include: arginine administration in vivo (Aguilar‐Parada et al., 1969; Gehrand et al., 2016); pronounced insulin‐induced hypoglycemia (Powell et al., 1993); or administration of 2‐deoxyglucose (Karlsson et al., 2002). Plasma concentrations are also markedly elevated in the hyperglucagonemia of rare alpha cell tumors (Batcher et al., 2011) or in glucagon receptor knockout mice (Gelling et al., 2003). Notably, in experimental studies of glucagon’s cellular mechanism of action, investigators often expose tissue or cellular preparations to 100,000 pM (usually expressed as 100 nM), an extreme zone 4 pharmacological concentration.

Bear in mind that the limits of these plasma glucagon concentration zones are based on estimates obtained by RIA, and as such they are very likely overestimates. “In normal physiology, circulating concentrations of glucagon are in the lower picomolar range. In the [usually overnight] fasting state with plasma glucose levels around 5 mmol/L, glucagon is secreted in basal levels resulting in plasma concentrations *below 20 pmol*/*L* [italics added]” (Rix et al., 2019). That conclusion was based on results of studies using C‐terminal‐directed RIA and ELISA‐based methods. ELISA assays tend to be more specific and sensitive than conventional RIAs are, and thus tend to yield lower values (Bak et al., 1985; Wewer Albrechtsen et al., 2014; Wewer Albrechtsen, Veedfald, et al., 2016). Peripheral venous plasma glucagon concentrations measured by RIA and ELISA are shown in Figure 3 and Table 1. Miyachi et al. (2017) compared three assay techniques for measuring human plasma glucagon levels: RIA, ELISA, and liquid chromatography/mass spectrometry (LC/MS) (Figure 3). They determined that peripheral venous samples taken from 13 volunteers, subjected to an overnight fast, yielded mean values of 24 pM by RIA, 12 pM by sandwich ELISA, and 8 pM by LC/MS. The ELISA/RIA concentration ratio of 50% is close to the ratio of 43% calculated from direct comparisons carried out in a group of studies listed in Table 1. Thus, the collective mean hepatic portal and peripheral venous plasma glucagon concentrations determined by RIA of 43.9 and 28.8 pM, respectively, as depicted in Figures 1 and 2b, might have been closer to 19 and 12 pM had they been measured by ELISA instead. The last is identical to the value of 12 pM reported for the ELISA‐based estimates obtained from peripheral venous plasma reported by Miyachi and coworkers. Peripheral venous plasma values of around 6 pM in the rat were reported by Xue, Cei, et al. (2017) using the Millipore RENDOI‐85K rat endocrine Linco‐plex assay, and in humans by Kobayashi (Kobayashi et al., 2020) using a sandwich ELISA assay. Based on the hepatic portal/peripheral venous concentration ratio of 1.5 depicted in Figure 2b, mean peripheral venous concentrations of 6–19 pM would translate to 9–29 pM in the hepatic portal circulation. It can thus be tentatively concluded that the mean hepatic portal plasma glucagon concentration in adult mammals including humans, under fed or short‐term fasting conditions at rest or during normal physical activity, may be anywhere from 9 to 29% of the AC‐activating TC, determined ex vivo, of 100 pM.

Seemingly, if it were any other hormone, such a substantial gap between normal physiological plasma concentrations and those required to generate the signal of interest would be sufficient grounds to rule out that signal as a mediator of the hormone’s effects. Indeed, Sutherland and coworkers, again in their 1971 paper (Exton, Robison, et al., 1971), applied that very logic to all but dismiss in vivo glucose‐mobilizing actions of two other hormones they looked at, the catecholamines epinephrine and norepinephrine. Their conjecture has withstood the test of time. The basal plasma epinephrine concentration range in rats or humans at rest is roughly 200–1,500 pM, but can increase 5‐ to 20‐fold during periods of elevated metabolic stress such as strenuous exercise (Carlson et al., 1985; Jean et al., 1991; Sellers et al., 1988; Winder et al., 1979, 1981, 1982), reaching peak levels as high as around 10,000 pM. By contrast, the epinephrine TC required to activate AC in Sutherland’s 1971 report was 28,000 pM. According to more recent reports, the TC can be as high as 100,000 pM (Noguchi et al., 1985; Pilkis & Ghranner, 1992; Pilkis et al., 1975; Yagami, 1995). Thus, even peak mean plasma epinephrine concentrations under stressful conditions are about 10 to 35% of the TC required to activate AC. Similar disparities apply to circulating norepinephrine as well. Sutherland et al. (Exton, Robison, et al., 1971) concluded: “… peak catecholamine concentrations [in dogs and humans] are lower than the minimal levels [TCs] for activation of cyclic AMP accumulation or glycogenolysis in the isolated rat liver…*[T]hese data would preclude circulating epinephrine or norepinephrine from physiological roles in the regulation of hepatic glucose output* [italics added]”. The authors were likely assuming that cAMP was the exclusive cellular signal mediating the glucose‐mobilizing actions of either glucagon or the catecholamines. They were of course unaware of the alternative signal discovered a decade and a half later, one that is activated by lower concentrations of either glucagon or the alpha agonist component of catecholamines (see the next section). Its alpha receptor‐activating property (Laville et al., 1987) accounts for the relatively low, physiological TC, 1000 pM, of epinephrine required to increase glucose output from rat hepatocytes (Bizeau & Hazel, 1999). The most important point here is that the disparities between mean plasma concentrations of epinephrine (under stress) or glucagon (under normal conditions) and their respective concentrations required to activate AC ex vivo are comparable, about 3‐ to 10‐fold for epinephrine and around 3‐ to 11‐fold for glucagon.

If that logic, as it applied to catecholamines, was valid for the scientists who discovered the signal, then the question remains: In light of a similar gap between plasma concentrations and those required to activate AC, why don’t most investigators apply the same criterion to glucagon and at least question the prevailing view that the AC/cAMP pathway is the major or exclusive mediator of its hepatic metabolic actions “most or all of the time”? In spite of strong evidence that they would be justified in doing so, most ignore the question altogether. The longstanding cAMP bias persists (e.g. Agius, 2007; Han et al., 2016; Janah et al., 2020; Jitrapakdee, 2012; Miller & Birnbaum, 2016; Wewer Albrechtsen et al., 2019; Yang & Yang, 2016; Zhang et al., 2019), despite the existence of a widely‐known, surprisingly well‐characterized, and much more physiologically relevant alternative signal.

The solution to the problem was discovered in 1986: There is more than enough glucagon in hepatic portal plasma to activate an alternative, physiologically relevant signal pathway to increase hepatic glucose mobilization.

## 4 DISCOVERY OF THE BASIC SIGNAL

In 1986, fifteen years after Sutherland and coworkers published their seminal report, Miles Houslay and colleagues published another landmark paper on hepatic actions of glucagon, this time on rat hepatocytes (Wakelam et al., 1986). Among their findings were: (1) Between the concentrations of 10 and 3000 pM, glucagon stimulated the production of inositol phosphates from membrane phospholipids dose‐dependently; (2) The TC of glucagon required to increase the production of cAMP was above 100 pM; and (3) A derivative of glucagon, 1‐N‐α‐trinitrophenylhistidine‐l2‐homoarginine (TH)‐glucagon, also increased the production of inositol phosphates, to a similar extent over a similar concentration range, without affecting cellular levels of cAMP. They proposed that glucagon activates two receptors, which they called GR1, coupled to inositol‐phosphate production, and GR2, coupled to AC. The existence of two glucagon binding sites in liver has been confirmed by direct receptor binding studies (Bonnevie‐Nielsen & Tager, 1983; Chamras et al., 1980; Ikezawa et al., 1998). It is now known that the high‐affinity, low‐density GR1 receptor is coupled to phospholipase C (PLC) and enhanced production of the biologically active inositol‐3,4,5‐triphosphate (IP3) (Goldstein & Hager, 2018; Müller et al., 2017; Rix et al., 2019). The low affinity‐high density GR2 receptor, linked to the increased production of cAMP as a product of AC activation (Agius, 2007; Han et al., 2016; Miller & Birnbaum, 2016; Yang & Yang, 2016) is by far the more widely studied.

Glucagon exerts dual effects on hepatic glucose mobilization. At physiological concentrations it submaximally stimulates glucose output solely by activating the PLC/IP3 pathway. At supraphysiological concentrations the hormone further stimulates glucose output by activating the PLC/IP3 and AC/cAMP pathways simultaneously.

### 4.1 Glucose output produced by activation of the two signal pathways

Studies carried out ex vivo show that glucagon promotes hepatic glucose mobilization by activating either pathway, but does so over different but overlapping concentrations ranges, as shown in Figure 4. The figure highlights several observations of importance. It clearly shows the disparity between the statistical physiological hepatic portal plasma concentration range, derived from the data in Figure 1, and the consensus TC for activating AC ex vivo, 100 pM, the result of a composite concentration‐effect curve generated from 15 studies published between 1971 and 1995. Within zone 1, the stimulation of glucose mobilization is associated with a submaximal but measurable and concentration‐dependent increase in the generation of inositol phosphates, with little or no activation of AC. According to the composite concentration‐effect curve generated by combining 11 studies published between 1971 and 1990, physiological, zone 1 glucagon concentrations stimulate glucose mobilization (gluconeogenesis, glycogenolysis, or glucose output) up to about 40% of the maximum, while activating the PLC/IP3 pathway exclusively. In zone 2, glucagon exerts variable effects on AC activity. Across zones 3 and 4, between 100 and 10,000 pM, glucagon maximally activates the PLC/IP3 pathway while sub‐maximally activating the AC/cAMP pathway and further increasing glucose mobilization. Maximal activation of the AC/cAMP pathway, with inhibition of the PLC/IP3 pathway, is achieved by high zone 4, pharmacological concentrations (Figure 4).

The dual effects of glucagon are incorporated into a proposed new model. Under normal conditions, glucagon regulates hepatic glucose mobilization exclusively via the PLC/IP3 pathway. During intervals of high systemic glucose demand, such as in the early neonate or strenuously exercising adult, glucagon at higher plasma concentrations boosts hepatic glucose output to meet the elevated glucose demand by activating both the PLC/IP3 and AC/cAMP pathways simultaneously.

Figure 4 clearly reveals the dual nature of glucagon’s glucose‐mobilizing actions on the liver. Below 60 pM, the hormone activates GR1 receptors and the PLC/IP3 pathway to stimulate glucose mobilization up to 40% of maximal capacity without activating AC. This corresponds to plasma concentrations that are characteristic of the normal, metabolically unstressed condition (Figure 2a and b). Above 100 pM, glucagon further stimulates glucose mobilization by activating both GR1 and GR2 receptors and the PLC/IP3 and AC/cAMP pathways simultaneously, applicable to the mean plasma concentrations found in the early neonate and exercising adult (Figure 2d). This suggests that, most of the time in vivo, glucagon regulates hepatic glucose output up to 40% of maximal capacity via the PLC/IP3 pathway exclusively. As plasma concentrations rise above 100 pM in response to elevations in systemic glucose demand, glucagon concentration‐dependently and adaptively boosts glucose output, by robustly activating the PLC/IP3 pathway toward its maximum and by submaximally activating the AC/cAMP pathway.

This dual effect mediated by two signal pathways seems to be a variation on a broader theme, applicable to target tissues other than hepatocytes. At a concentration of 10,000 pM, glucagon increased cAMP in rat renal glomerular mesangial cells (Li et al., 2006). The same concentration also increased calcium mobilization, an effect that was totally blocked by the PLC inhibitor U73122, implicating the PLC/IP3 pathway. At 1000 pM, sufficient to maximally increase prostaglandin production, glucagon markedly increased both cAMP and inositol phosphates in hepatic Kupffer cells (Hespeling et al., 1995). As in hepatocytes, the operative signal in Kupffer cells most of the time is probably the PLC/IP3 pathway alone because the stimulation of prostaglandin production was maximal or near‐maximal at 100 pM. Human adipose tissue is nearly or completely unresponsive to lipolytic effects of glucagon, even at zone 4 concentrations (Galsgaard et al., 2019; Rodgers, 2012). Recently, however, (Pereira et al., 2020) reported that glucagon concentration‐dependently but weakly enhanced glycerol release by human hepatocytes, with a TC of 10 pM. The effect of that low physiological concentration on glycerol release was statistically significant but one of a similar magnitude on glucose uptake was not because it was too variable. It seems that the physiological receptor and signal mediating effects of glucagon at physiological concentrations on human adipocytes remain to be identified. Glucagon may also activate two signals in the heart (Harney & Rodgers, 2008). In addition to activating the AC/cAMP pathway to increase contractility at supraphysiological concentrations, at or above 300 pM, glucagon at physiological concentrations, between 10 and 80 pM, also substantially enhanced cardiac glucose utilization by interacting with an uncharacterized receptor coupled to PI3‐kinase, presumably with only the latter operative in vivo under normal conditions (Harney & Rodgers, 2008; Rodgers, 2012). Although glucagon clearly works against insulin on the liver to promote glucose output, it may also partner with insulin on the heart to stimulate glucose uptake and utilization. In the following sections, hepatic glucose‐mobilizing effects of glucagon that are mediated exclusively by activation of GR1 receptors and the PLC/IP3 pathway at physiological concentrations are presented, along with examples of overlapping cellular responses to activation of the AC/cAMP pathway by higher concentrations.

The two intracellular signal pathways activated by glucagon in hepatocytes are largely redundant. Many of the downstream targets of the PLC/IP3 pathway are the same as those of the AC/cAMP pathway.

## 5 INTRACELLULAR EFFECTS OF ACTIVATING THE PLC/IP3 signal

**FIGURE 5:**
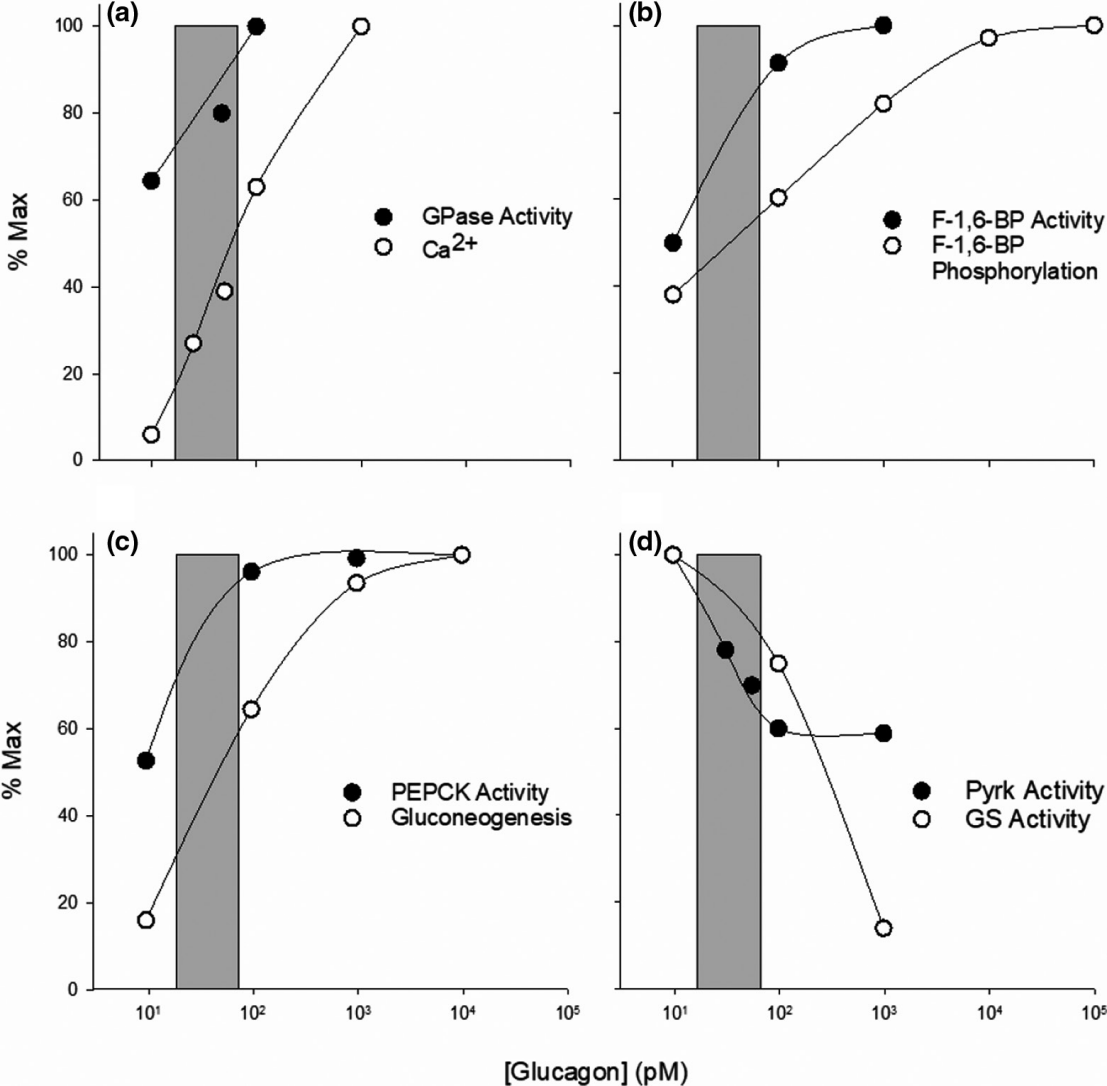
Comparisons of glucagon concentration‐effect curves, generated in rat hepatocyte preparations, for selected components and targets of the PLC/IP3 pathway. (a) glycogen phosphorylase (GPase) activity and intracellular calcium levels (Ca^2+^_i_); (b) fructose‐1,6‐bisphosphatase (F‐1,6‐BP) activity and phosphorylation; (c) phosphoenolpyruvate carboxykinase (PEPCK) activity and gluconeogenesis; and (d) activities of pyruvate kinase (PyrK) and glycogen synthase (GS). The shaded vertical bars represent the statistical range of plasma glucagon concentrations in the hepatic portal system (see legends of Figures 1 and 4). At the upper limit, mediated exclusively by the PLC/IP3 pathway, glucagon increased Ca^2+^i 68% and GPase activity 93% (a), F‐1,6‐BPase activity 87% and its phosphorylation 53% (b), and PEPCK expression (activity) 94% and gluconeogenesis 57% (c) of the maximum response produced by higher concentrations. It also decreased PyrK activity by 37% and GS activity by 19% (d). Effects produced by concentrations above 100 pM are mediated by the activation of both the PLC/IP3 and AC/cAMP pathways simultaneously. The data are adapted from Aggarwal et al. (1995), Ekdahl and Ekman (1987), Fleig et al. (1984), Marks and Parker Botelho, (1986) and Staddon and Hansford (1989). See also El‐Maghrabi et al. (1982), Felíu et al. (1976), Ikezawa et al. (1998) and Studer et al. (1984). The data are consistent with the hypothesis that glucagon, at physiological concentrations, exerts substantial effects on intracellular targets involved in glucose mobilization by activating the PLC/IP3 pathway without activating the AC/cAMP pathway

Activation of the PLC/IP3 pathway at physiological concentrations generates many of the same intracellular effects as does the activation of the AC/cAMP pathway at supraphysiological and pharmacological concentrations. Downstream targets of the two pathways overlap extensively. Common intracellular effects include increases in calcium concentrations and altered activities and expressions of the same enzymes. In the following discussion, downstream targets of the PLC/IP3 pathway are described in some detail, along with examples of similar or identical targets or effects of activating the AC/cAMP pathway (Table 2, Figures 4 and 5).

**TABLE 2:** Threshold concentrations (TCs) or effective single concentrations of glucagon sufficient to activate the GR1/PLC/IP3/Ca/CaM signal and downstream events

Response	TC or Effective concentration	References
	(pM)	
A. Signal activation and calcium mobilization		
↑ PLC and inositol‐P production	5	Wakelam et al. (1986)
↑ Ca^2+^_i_ or spike frequency	17	Berglund et al. (2009), Kass et al. (1994), Mine et al. (1988), Sistare et al. (1985), Somogyi et al. (1992) and Staddon and Hansford (1989)
B. Cytosolic enzyme targets		
↑ GPase activity	13	Blackmore and Exton (1986), Lynch et al. (1989), Marks and Parker Botelho (1986), Rothermel, Perillo, et al. (1984) and Shiota et al. (1996)
↑ F−1,6‐BP phosphorylation	10	Aggarwal et al. (1995)
↑ F−1,6‐BP activity	6	Caruana et al. (1981) and Ekdahl and Ekman (1987)
↑ F−2,6‐BP activity	5	El‐Maghrabi et al. (1982)
↓ 6‐PF−2K activity	5	El‐Maghrabi et al. (1982)
↑ PyrK phosphorylation	10	Aggarwal et al. (1995)
↓ PyrK activity	40	Blair et al. (1976), El‐Maghrabi et al. (1982), Felíu et al. (1976)
↑ GS phosphorylation	1	Aggarwal et al. (1995)
↓ GS activity	6	Marks and Parker Botelho (1986) and Rothermel, Jastor, et al. (1984)
↑ AMPK phosphorylation	**47**	Kraft et al. (2017) and Rivera et al. (2010)
↑ ACC phosphorylation	**57**	Rivera et al. (2010)
C. Nuclear targets		
↑ PFKFB1 phosphorylation	10	Miller et al. (2013)
↑ Fox01 phosphorylation	**57**	Rivera et al. (2010)
↑ CREB phosphorylation	10	Miller et al. (2013)
↑ PEPCK expression	1	Fleig et al. (1984)
↓ GK expression	**34**	Kraft et al. (2017)
D. Net responses		
↓ Glycogen synthesis	**34**	Kraft et al. (2017)
↑ Glycogenolysis	9	Bizeau and Hazel (1999; Corvera and García‐Sáinz (1984), Exton, Lewis, et al. (1971), Khan et al. (1980), Rothermel, Jastor, et al. (1984), Yamatani et al. (1987)
↑ Gluconeogenesis	23	Exton, Lewis, et al. (1971), Felíu et al. (1976), Fleig et al. (1984) and Shiota et al. (1996)
↑ Glucose output	20	Fleig et al. (1984) and Shiota et al. (1996)
Mean ± SEM (*n* = 17)	11.2 ± 2.3	

Note

The TCs were determined from dose‐response curves generated in hepatocytes or perfused livers. Effective single concentrations (bold) were achieved by infusions of exogenous hormone in vivo (dogs or mice). The values for F‐2,6‐BP and 6‐PF‐2K activities are estimates based on interpolations of the dose‐response curves. Where more than one reference is cited for a given response, the indicated TC is the group average. Calculation of the mean was restricted to the TCs determined from dose‐response curves ex vivo; single plasma concentrations generated by infusions in vivo were not included. Abbreviations: ACC, acetyl CoA carboxylase; AMPK, AMP‐activated protein kinase; F‐1,6‐BP, fructose‐1,6‐bisphosphatase; F‐2,6‐BP, fructose‐2,6‐bisphosphatase; 6‐PF‐1K, 6‐phosphofructo‐1‐kinase; GK, glucokinase; GPase, glycogen phosphorylase; GS, glycogen synthase; PEPCK, phosphoenolpyruvate carboxykinase; PFK, phosphofructokinase; PK, pyruvate kinase.

### 5.1 Signal activation and calcium mobilization

Activation of either glucagon GPCR isoform increases intracellular calcium (Ca^2+^_i_), but the mechanisms are not identical. The two glucagon GPCRs in liver, GR1 and GR2, are coupled to the G protein isoforms Gαq and Gαs, respectively (Christophe, 1995). Over a broad concentration range (Figure 4), glucagon binds to the high‐affinity, low density GR1 GPCR (Andersson et al., 1993; Bonnevie‐Nielsen & Tager, 1983; Chamras et al., 1980; Ikezawa et al., 1998) and activates PLC and the formation of phospholipid products including the biologically active inositol‐1,4,5‐triphosphate (IP3) (Hansen et al., 1982; Unson et al., 1989; Wakelam et al., 1986; Whipps et al., 1987). Binding of IP3 to inositol triphosphate receptors (IP3Rs) releases reticular calcium while generating calcium spikes and oscillations (slower waves) that propagate throughout the cell and increase the total Ca^2+^_i_ levels in the cytosol and nucleus (Aromataris et al., 2006; Bartlett et al., 2014; Bill & Vines, 2020; Bygrave & Benedetti, 1993; Hernandez et al., 2007; Hirata et al., 2002; Kass et al., 1994; Klein & Malviya, 2008; Kraus‐Friedmann, 1986; Reber et al., 1990; Thomas et al., 1991; Whipps et al., 1987) (Table 2A), (Table 2A, Figure 5).

There are two isoforms of the IP3R in hepatocytes, IP3R‐I and ‐II (Hernandez et al., 2007; Hirata et al., 2002; Mauger et al., 1989). Both are located on reticular elements within the cytosol, but the distribution of the IP3R‐I receptor is greater, extending into the nucleus (Feriod et al., 2014; Hirata et al., 2002; Klein & Malviya, 2008). IP3R‐II receptors are concentrated proximal to the canalicular membrane. Increases in Ca^2+^_i_ produced by GR1 activation then bind to CaM, forming a Ca^2+^/CaM complex. The complex binds to and activates multiple isoforms of Ca^2+^/CaM‐dependent protein kinases (collectively labelled here as CaMK). CaMKs implicated in the glucose‐mobilizing actions of glucagon in liver include CaMKII, CaMKIV, and CaMKKβ (aka CaMKK2) (Cohen et al., 2015; Hook & Means, 2001; Johannessen & Moens, 2007; Shaywitz & Greenberg, 1999; Skelding & Rostas, 2020; Soderling, 1999; Takemoto‐Kimura et al., 2017). Apparently, only the IP3R‐I isoform mediates the effects of calcium‐mobilizing hormones such as vasopressin (Hernandez et al., 2007) or, presumably, glucagon via GR1 activation to increase Ca^2+^_i_ and generate related downstream effects. Liver‐specific knockout of the IP3R‐I receptor prevented the increase in CaMKII phosphorylation and other responses to high concentrations of glucagon (Perry et al., 2020). Influences on physiological concentrations were not tested. Knockout of the IP3R‐II receptor had little to no effect on glycemia, gluconeogenesis, fasting‐induced activation of CaMKII, increases in the expressions of the gluconeogenic enzymes phosphoenolpyruvate carboxykinase (PEPCK) or glucose‐6‐phosphatase (G6Pase) in response to 50,000 pM glucagon, or on the locations or densities of IP3R‐I receptors (Feriod et al., 2014).

Mobilization of intracellular calcium is strongly affected by physiological concentrations of glucagon. The TC required to increase Ca^2+^_i_ in hepatocytes is most often reported to be 10 pM, a low zone 1 concentration (Kass et al., 1994; Sistare et al., 1985; Staddon & Hansford, 1989). Within zone 1, activation of the PLC/IP3/IP3R pathway by glucagon increases Ca^2+^_i_ up to approximately 55% of the peak response generated by maximal concentrations of the hormone (Figure 5a). The ability of 50 nM glucagon to produce calcium waves and increase Ca^2+^_i_ was completely blocked by the PLC antagonist U73312 (Aromataris et al., 2006). Like low concentrations of glucagon, the calcium‐mobilizing hormones angiotensin, vasopressin, or norepinephrine increase Ca^2+^_i_ and induce calcium waves by activating PLC without activating AC (Charest et al., 1985; Hernandez et al., 2007; Pittner & Spitzer, 1993; Ubl et al., 1994; Woods et al., 1986). In the presence of sub‐TCs of the alpha agonist phenylephrine, glucagon evoked calcium spikes or oscillations (waves superimposed by repetitive spikes) in rat hepatocytes at concentrations as low as 4 pM (Somogyi et al., 1992), suggesting that the effects of the two hormones are additive.

The dual nature of glucagon’s hepatic effects is represented well by its influence on intracellular calcium. In addition to activating the PLC/IP3 pathway, activating the AC/cAMP pathway also increases Ca^2+^_i_, but by a slightly different mechanism. At concentrations within zone 3 or higher, sufficient to consistently activate PKA, glucagon adds to the IP3‐induced increase in Ca^2+^i by stimulating PKA‐mediated phosphorylation of both reticular IP3Rs and plasma membrane calcium channels (Andersson et al., 1993; Joseph & Ryan, 1993; Wang et al., 2012; Williamson et al., 1987). Thus, at physiological concentrations, glucagon mimics the effects of calcium‐mobilizing hormones to increase Ca^2+^_i_ by activating the GR1/PLC/IP3/CaM pathway exclusively, promoting Ca^2+^ movement into the cytosol by increasing the binding of IP3 to reticular IP3Rs. At supraphysiological concentrations glucagon further increases Ca^2+^_i_ by activating both the GR1/PLC/IP3 and GR2/AC/cAMP/PKA pathways simultaneously, the latter adding to the movement of calcium into the cytosol by PKA‐mediated phosphorylation of reticular IP3Rs and plasma membrane calcium channels.

Both pathways also seem to be involved in the control of ion flux across the plasma membrane. For example, infusion of anti‐glucagon antibodies diminishes chronic hyperpolarizing actions of endogenous glucagon on the liver (Lutz et al., 2001). The hyperpolarizing effect of 100,000 pM glucagon in hepatocytes is inhibited approximately 60% by 1 μM U73122 (Fischer et al., 2005). The residual response in the presence of the high PLC blocker concentration is consistent with the view that alterations of plasmalemmal ion channel conductance are produced by activation of either pathway. Specific plasma membrane ion channels that are targeted by physiological concentrations of glucagon apparently remain to be identified, but both calcium and chloride channels are implicated. At low extracellular calcium, glucagon concentration‐dependently promoted net efflux of calcium, at or above 10 pM (Mauger et al., 1989). Vasopressin (10,000 pM) produced a similar response. However, when extracellular calcium concentration is in the normal range, glucagon (10–100 pM) promotes its influx and adds to analogous effects of epinephrine, presumably the alpha agonist component (Poggioli et al., 1986). Activation of plasmalemmal chloride channels by 50,000 pM glucagon was completely blocked by 4 μM U73122 (Aromataris et al., 2006).

Because both glucagon—at physiological concentrations—and the calcium‐mobilizing hormones (e.g., norepinephrine, vasopressin, and angiotensin II) act via the GR1/PLC/IP3 pathway, their effects on glucose mobilization should be similar or identical. However, comparisons are usually made using high concentrations of both calcium‐mobilizing hormones, which presumably activate the PLC/IP3 pathway exclusively regardless of concentration, and glucagon, which activates both the PLC/IP3 and AC/cAMP pathways simultaneously at high concentrations. As a result, the actions of calcium‐mobilizing hormones and glucagon, at higher concentrations at least, are often different. Common downstream targets of, and cross‐talk between, the overlapping pathways complicates the interpretation of effects of high glucagon concentrations (Goldstein & Hager, 2018; Jiang & Zhang, 2003; Müller et al., 2017; Rix et al., 2019). For example, at their respective maximal concentrations, glucagon (570,000 pM) tripled the basal activity of glycogen phosphorylase (GPase), but vasopressin (200,000 pM), angiotensin II (100,000 pM), or phenylephrine (1,000,000 pM) only increased it 2‐ to 2.5‐fold in human hepatocytes (Keppens et al., 1993). The differences in this case can be at least partially explained by the ability of higher glucagon concentrations to activate the reticular IP3R two ways as discussed above, one by IP3 binding—like the effects of the calcium‐mobilizing hormones—and a supplementary effect produced by PKA‐mediated phosphorylation of the IP3R. Other examples of pathway crosstalk points include: CaMKK and CaMKII, which are activated by physiological glucagon concentrations via the PLC/IP3 pathway but can also be phosphorylated by PKA at higher concentrations, inhibiting the activity of the former and stimulating the activity of the latter (Ozcan et al., 2012; Racioppi & Means, 2012; Skelding & Rostas, 2020); AC, which can be inhibited by CaMKIV (Soderling, 1999); and, as discussed below, PhosK, which can be activated either by elevations in Ca^2+^i (as a result of activation of either pathway) or by PKA‐mediated phosphorylation of the enzyme.

To complicate the picture further, the hepatic effects of calcium‐mobilizing hormones are subtly different from each other (Kleineke & Söling, 1987) as well as from those of physiological concentrations of glucagon, even though all of these agonists presumably act via the same intracellular signal pathway. Differences are revealed by variations in effects of phorbol ester pretreatment and, in a mechanistically related phenomenon, the extent and kinetics of rapid homologous desensitization. Activation of the PLC/IP3 pathway can generate diacylglycerol (DAG), which binds to and activates PKC, an effect mimicked by phorbol esters (Newton, 2018; Nishizuka, 1989; Püschel et al., 1993). Activated PKC suppresses inositol‐phosphate generation by directly inhibiting PLC and by increasing the activity of inositol‐phosphate phosphatase (Higashi & Hoek, 1991; Savage et al., 1995; Williamson et al., 1987; Wu‐Zhang & Newton, 2013), exerting feedback inhibition of IP3 generation. This mechanism can explain both inhibitory effects of phorbol esters and rapid homologous desensitization exhibited by some—but not all—activators of the PLC/IP3 pathway. Phorbol esters have been reported to inhibit the glucose‐mobilizing actions of alpha agonists, but not of vasopressin or angiotensin II (Corvera & García‐Sáinz, 1984). Pretreatment with the phorbol ester PMA inhibited the stimulation of GPase by the α‐agonist component of epinephrine and by 10–1000 pM vasopressin, but did not affect the dose‐dependent activation of the enzyme by 10–1000 pM glucagon in rat hepatocytes (Lynch et al., 1985). As predicted, pretreatment with PMA suppressed the increase in glucose output from perfused rat livers produced by norepinephrine but it did not alter the response to 10,000 pM glucagon (Püschel et al., 1993). In rat hepatocytes, the generation of inositol phosphates or the stimulation of GPase by vasopressin faded within 10 min (Hughes et al., 1984), presumably as a result of DAG‐induced activation of PKC. However, either glucagon or TH‐glucagon (the selective GR1 agonist) produced a sustained increase in inositol‐phosphate generation over that same time period (Wakelam et al., 1986), suggesting that the PLC/IP3‐dependent pathway activated by glucagon via the GR‐1 receptor is not subject to rapid DAG‐mediated desensitization. Disparate responses may be explained, at least partially, by differences in G protein isoforms coupled to their receptors or in fatty acyl groups bound to the DAG molecules generated by the different agonists (Hughes et al., 1984; Morel et al., 1992; Wu‐Zhang & Newton, 2013; Xu & Xie, 2009).

### 5.2 Cytosolic metabolic enzyme targets

Activation of the PLC/IP3 pathway alters activities of key cytosolic and mitochondrial enzymes involved in the regulation of glucose metabolism and mobilization (Table 2B), many of which are also altered in the same direction by PKA. Important direct and indirect targets are phosphorylase kinase (PhosK), glycogen phosphorylase (GPase), fructose‐1,6‐bisphosphatase (F‐1,6‐BPase), fructose‐2,6 bisphosphatase (F‐2,6‐BPase), pyruvate kinase (PyrK), phosphofructokinase‐1 (PFK‐1), glycogen synthase (GS), and 5’‐AMP‐activated protein kinase (AMPK). Activation of either the PLC/IP3/CaM or the AC/cAMP/PKA pathway generally targets the same enzymes and alters their activities in the same direction, but there are differences in detail (Table 2B).

Another good example of the dual action of glucagon is the control of the activity of PhosK, which phosphorylates and activates GPase (Brushia & Walsh, 1999; Miller & Birnbaum, 2016; Rothermel et al., 1984; Shiota et al., 1996), promoting glycogenolysis. PhosK is activated by elevations in Ca^2+^_i_, which as mentioned earlier can be produced by glucagon in response to activation of either pathway (Roskoski, 2015). At physiological concentrations glucagon increases Ca^2+^_i_ by activating the GR1/PLC/IP3/CaM pathway, promoting IP3 binding to reticular IP3Rs and the movement of Ca^2+^ into the cytosol. In addition, at supraphysiological concentrations glucagon can further increase Ca^2+^i by direct PKA‐mediated phosphorylation of IP3Rs and of plasmalemmal calcium transporters in response to the activation of GR2 receptors (Roskoski, 2015). Elevations in Ca^2+^i produced by either mechanism activate PhosK by increasing the extent of calcium’s binding to the CaM component of the enzyme (Vénien‐Bryan et al., 2002). Activated PhosK then converts GPase **b** to the active GPase **a** by catalyzing its phosphorylation. The end result of the activation of either pathway is increased glycogenolysis and enhancement of glucose output. One implication is that, in vivo, variations in plasma glucagon within the physiological range would regulate hepatic glycogenolysis at sub‐maximal levels by activating GR1 receptors exclusively. Higher plasma concentrations could then enhance the stimulation of glycogenolysis by activating both GR1 and GR2 receptors.

There are other examples of overlap between the pathways with regard to common target enzymes. Either CaMK or PKA catalyzes the phosphorylation of the bifunctional enzyme 6‐phosphofructo‐2‐kinase (6‐PF‐2K or PFK2)/fructose‐2,6‐bisphosphatase (F‐2,6‐BPase), increasing the activity of the phosphatase component (Brushia & Walsh, 1999; El‐Maghrabi et al., 1982; Miller & Birnbaum, 2016; Okar et al., 2004; Ravnskjaer et al., 2015). This would decrease levels of the allosteric regulator fructose‐2,6‐bisphosphate (F‐2,6‐BP). In the classical view, declines in F‐2,6‐BP would lift inhibition of the gluconeogenic enzyme FBP‐1 and decrease the activation of the glycolytic enzyme PFK‐1, promoting gluconeogenesis while inhibiting glycolytic flux (El‐Maghrabi et al., 1982; Exton, 1987; Furuya et al., 1982; Mlekusch et al., 1981; Müller et al., 2017; Okar et al., 2004). In addition, either CaMK or PKA can phosphorylate the gluconeogenic enzyme F‐1,6‐BPase in vitro (Mlekusch et al., 1981) or in hepatocytes, increasing its activity (Casteleijn et al., 1986; Ekdahl & Ekman, 1987; El‐Maghrabi et al., 1982). Activation of either pathway can phosphorylate and decrease the activity of the glycolytic enzyme PyrK (Blair et al., 1976; Connelly et al., 1987; Felíu et al., 1976; Mlekusch et al., 1981; Miller & Birnbaum, 2016; Staddon & Hansford, 1989), providing an additional mechanism of promoting gluconeogenesis and inhibiting glycolysis. Glycogen synthase (GS) is a promiscuous target. It can be phosphorylated and inhibited not only by PKA or CaMK, but also by PhosK, PKC, and glycogen synthase kinase, among others (Aggarwal et al., 1995; Camici et al., 1984; Ciudad et al., 1984; Imazu et al., 1984; Juhl et al., 1983; Marks & Parker Botelho, 1986; Rothermel et al., 1984; Staddon & Hansford, 1989; Wang et al., 1986).

Adenosine monophosphate‐activated protein kinase (AMPK) is an important metabolic sensor that responds to variations in intracellular AMP/ATP ratios and other regulators to balance anabolic and catabolic cellular processes (Aw et al., 2014; Berglund et al., 2009; Fullerton, 2016; Mihaylova & Shaw, 2011; Salminen et al., 2016; Zhang, Yang, et al., 2019). Glucagon affects its activity exclusively via the PLC/IP3 pathway. Activated CaMKKβ, but not PKA, catalyzes the phosphorylation and activation of AMPK (Fujiwara et al., 2016; Fullerton, 2016; Racioppi & Means, 2012; Witters et al., 2006). However, it is not clear how or even whether phosphorylation and activation of AMPK via CaMKKβ contributes to the resulting increase in glucose output produced by physiological concentrations of glucagon. The net response may depend on such attendant influences as actions of other hormones and regulators, net flux through multiple enzyme pathways, indirect influences on glucose metabolism imposed by AMPK‐mediated alterations in fatty acid metabolism, and net changes in the cellular AMP/ATP ratio and energy state (Aw et al., 2014; Berglund et al., 2009; Fullerton, 2016; Hasenour et al., 2013; Marcelo et al., 2016; Mihaylova & Shaw, 2011; Perry et al., 2020; Willows et al., 2017).

Activated AMPK is often depicted as an inhibitor of gluconeogenesis (Mihaylova & Shaw, 2011). Liver‐specific overexpression of AMPK was reported to inhibit glucose output, resulting in hypoglycemia (Foretz et al., 2019). Phosphorylation of CRTC2 by AMPK suppresses translocation of CRTC2 to the nucleus and, by that mechanism, inhibits gluconeogenesis (Yoshida et al., 2019). Pharmacological activation of AMPK in hepatoma cells mimicked effects of insulin to decrease the expressions of the gluconeogenic enzymes PEPCK and G6Pase (Lochhead et al., 2000). Constitutively active AMPKα1 decreased PEPCK expression, while dominant negative AMPKα1 increased PEPCK expression in mouse liver (Viana et al., 2006).

But other results suggest that, under some circumstances, activation of AMPK may contribute to the stimulation of gluconeogenesis and glucose output by glucagon. Starvation can increase plasma glucagon (Figure 2c) and has been reported to increase AMPK activity while also increasing gluconeogenesis and decreasing glycogen synthesis (Munday et al., 1991; Sugden et al., 1993). Hepatocytes from CaMKKβ knockout mice exhibited decreased expressions of G6Pase and PEPCK and released approximately 50% less glucose when compared to hepatocytes from wild type mice (Anderson et al., 2012). Exercising rats displayed increases in plasma glucagon (from 19 to 49 pM), hepatic AMPK activity (by 73%), and plasma glucose (by 17%) (Carlson & Winder, 1999).

A substrate of AMPK is acetyl CoA carboxylase (ACC), which catalyzes the conversion of acetyl CoA to malonyl CoA, an intermediate in the lipogenic pathway (Hasenour et al., 2013; McGarry & Brown, 1997). ACC is phosphorylated and inhibited by activated AMPK. The inactivation of ACC by a high concentration of glucagon (100,000 pM) was reported to be independent of PKA, but instead was presumably the result of activation of CaMKKβ and AMPK (Sim & Hardie, 1988). The increase in AMPK activity in exercising rats (Carlson & Winder, 1999) was associated with a reduction of ACC activity of about 66%. Activation of the PLC/IP3/CaM pathway by glucagon has also been proposed to enhance gluconeogenesis indirectly by promoting lipolysis and mitochondrial β oxidation of fatty acids (Perry et al., 2020).

### 5.3 Nuclear effects

The dual actions of glucagon are also represented by its concentration‐dependent transcriptional effects (Table 2C). PEPCK catalyzes the conversion of oxaloacetate to phosphoenolpyruvate, a rate‐limiting step in gluconeogenesis. G6Pase is the final step in the gluconeogenic pathway, catalyzing the dephosphorylation of glucose‐6‐phosphate, allowing the export glucose from the liver to the bloodstream (Warner et al., 2020). Indirect and direct evidence suggest that either activation of the PLC/IP3/CaM pathway at physiological concentrations or the simultaneous activation of the PLC/IP3/CaM and AC/cAMP/PKA pathways at higher concentrations increases hepatic gluconeogenesis and glucose mobilization to a great extent by CaMK‐ or PKA‐dependent stimulation of the expressions and thus the activities PEPCK and G6Pase (Barthel & Schmoll, 2003; Hansen & Reshef, 1997; Johannessen & Moens, 2007). Key signal targets and coregulators involved in the transcriptional regulation of gluconeogenic gene expression include cyclic AMP response element binding protein (CREB), forkhead box class 01 protein (Fox01), CRTC2 (aka TORC2), and peroxisome proliferator‐activated receptor‐gamma coactivator alpha (PGC‐1α) (Oh et al., 2013; Zhang, Yang, et al., 2019). Phosphorylation of CREB and Fox01, and dephosphorylation of CRTC2, enhance the expressions of G6Pase and PEPCK, promoting gluconeogenesis (Anyamaneeratch et al., 2015; Li et al., 2019; Oh et al., 2013; Ozcan et al., 2012; Servillo et al., 2002; Wang et al., 2009). CREB can be phosphorylated by CaMKII, CaMKIV, or PKA in vitro (Shaywitz & Greenberg, 1999). CaMKII, CaMKIV, and PKA can all catalyze the phosphorylation of CREB at the ser‐133 residue of the kinase‐inducible domain, but CaMKII can also phosphorylate it at ser 142, 143, and 156 (Johannessen & Moens, 2007; Shaywitz & Greenberg, 1999). Thus, activation of either pathway by glucagon can theoretically promote phosphorylation of CREB via activation of CaMKII and CaMKIV at physiological concentrations or of CaMKII, CaMKIV, and PKA at supraphysiological concentrations in intact cells (Johannessen & Moens, 2007; Koo et al., 2005; Ravnskjaer et al., 2015). Interestingly, in the nucleus CaMKIV is more potent than CaMKII in phosphorylating CREB (Sun et al., 1994), and apparently can phosphorylate and activate CREB as effectively as PKA does (Soderling, 1999) (Table 2C).

Direct and indirect evidence indicate that physiological, zone 1 concentrations influence PEPCK expression via the PLC/IP3 pathway. In mouse liver, exogenous glucagon increased phosphorylation and activation of CREB at an apparent TC of 30 pM (Miller et al., 2013), most likely by ultimately activating CaMKIV (Cohen et al., 2015; Liu et al., 2020). Injection of glucagon (5 μg/Kg) into mice increased CREB phosphorylation, but neither the phosphorylation site nor the effect of the injection on plasma glucagon levels was specified (Koo et al., 2005). Ozcan et al., 2012 reported that fasting‐induced activation P38 and CaMKII increased phosphorylation of Fox01 and its subsequent migration to the nucleus, but did not measure parallel changes in plasma glucagon levels. Even against a background of sustained plasma insulin levels, increasing plasma glucagon concentration within zone 1, from around 13 to between 40 and 57 pM, by exogenous glucagon infusion in overnight‐fasted dogs increased FoxO1 and CREB phosphorylation and PEPCK expression, while decreasing liver glycogen content, glucose incorporation into glycogen, and liver glucose uptake (Kraft et al., 2017; Rivera et al., 2010).

Regulation by glucagon of G6Pase expression at physiological concentrations in vivo is not as clearly defined as it is for higher concentrations ex vivo, sufficient to also activate PKA consistently. The evidence is incomplete and conflicting. Liver‐specific CaMKKβ (CaMKK2) knockout mice expressed less hepatic G6Pase than the wild types did (Anderson et al., 2012). Although fasting for 6 h increased plasma glucagon levels into zone 2, from 18 to 87 pM, it did not alter hepatic G6Pase activity in mice (Mutel et al., 2011). However, STZ‐induced diabetes of 20 weeks duration in mice increased mean plasma glucagon from 13 to 78 pM and increased the expression of G6Pase three‐fold (Zhang et al., 2018). Starvation (72 h) of 2‐month‐old rats had no effect on plasma glucagon levels but depleted hepatic glycogen and reduced hepatic G6Pase and PEPCK activities (Bois‐Joyeux et al., 1986). Neither the intracellular levels of inositol‐phosphate nor those of cAMP was reported in any of these studies.

The phosphatase calcineurin is Ca^2+^/CaM‐dependent (Chin & Means, 2000), and seems to be involved in the translocation of signal components into the nucleus. Increasing Ca^2+^_i_ by activation of either pathway favors the formation of a Ca^2+^/CaM‐calcineurin complex, increasing its phosphatase activity (Chin & Means, 2000; Hook & Means, 2001). Two important substrates of activated calcineurin are cytosolic CaMKII and CRTC2. Phosphorylation of CaMKII by activated calmodulin CaMKK enhances its activity and traps it and its substrate, CRTC2, in the cytosol (Cohen et al., 2015). Dephosphorylation of both CaMKII and CRTC2 by Ca/CaM‐activated calcineurin promotes the translocation of CaMKII and CRTC2 into the nucleus. Studies of neurons suggest that, within the nucleus, Ca/CaM‐ activated CaMKII then activates CaMKIV, by direct interaction and via CaMKK (Cohen, 1989; Cohen et al., 2015). Activated CaMKIV then phosphorylates and activates CREB, promoting gluconeogenic gene transcription. In the liver, phosphorylation of cytosolic Fox01 by CaMKII via P38 has been proposed to promote its translocation into the nucleus to contribute to the enhancement of gluconeogenic gene transcription (Anyamaneeratch et al., 2015; Cohen et al., 2015; Erion et al., 2013; Ozcan et al., 2012).

In this context, the overlap between the two pathways in response to higher glucagon concentrations presents problems of data interpretation. For example, in mice starved for 24 h, 100,000 pM glucagon increased phosphorylation of IP3 receptors, a PKA‐specific effect (Wang et al., 2012). It also increased Ca^2+^_i_, an effect of activating both pathways at that high concentration. The elevated Ca^2+^_i_ stimulated the CaM‐dependent phosphatase calcineurin and consequent dephosphorylation of CREB‐regulated transcription coactivator 2 (CRTC2), promoting its migration to the nucleus and its contribution to the enhancement gluconeogenic gene expression. Although it is very likely that both pathways were activated and involved in producing the ultimate response, the authors interpreted their results strictly within the context of the activation of the AC/cAMP/PKA pathway alone. It would be interesting if similar results, albeit perhaps at a lower magnitude, might be achieved in response to glucagon at 60 pM, presumably in response to activation of only the PLC/IP3 pathway.

### 5.4 Net effects on glucose output

Under normal conditions, the contributions of glycogenolysis and gluconeogenesis to hepatic glucose production are approximately equal. That balance is tipped toward gluconeogenesis by fasting or starvation but variably altered by diabetes or exercise (Nordlie et al., 1999; Warner et al., 2020). The resulting transcriptional and post‐translational responses to activation by glucagon of the PLC/IP3/Ca/CaM pathway at physiological concentrations – and of the AC/cAMP/PKA pathway at higher concentrations ‐ is an elevation in either glycogenolysis or gluconeogenesis and in net glucose output (Table 2D). Relationships between physiological glucagon concentrations and changes in selected intracellular targets of the PLC/IP3 pathway are shown in Figure 5. Between 27.8 (10^1.44^) and 60.0 (10^1.78^) pM, the statistical physiological hepatic portal glucagon concentration range illustrated in Figure 1, glucagon increased: Ca^2+^_i_ up to approximately 50% and GPase activity to 90% (Figure 5a); F‐1,6‐BPase activity to 85% and its phosphorylation to 55% (Figure 5b), the expression and thus the activity of the gluconeogenic enzyme PEPCK to 70%, and gluconeogenesis to 60% of the maxima generated by higher concentrations (Figure 5c). It also inhibited the activities of PyrK and GS up to 40 and 90%, respectively (Figure 5d). The responses of these downstream effects to 60 pM correspond to a net increase in glucose output of approximately 40% of the maximum (Figure 4). These independent studies provide further evidence that glucagon, at plasma concentrations that prevail most of the time in vivo, even in type 2 diabetes, produces robust intracellular effects leading to enhanced glucose output by activating the PLC/IP3 pathway without activating the AC/cAMP pathway. This conclusion would be further supported by demonstrating substantial gaps between minimal concentrations required to generate glucose‐mobilizing responses, by activating the PLC/IP3 pathway, and higher TCs required to activate the AC/cAMP pathway, measured in the same study to avoid between‐laboratory variability (Table 2D, Figure 5).

### 5.5 Threshold concentrations of glucagon required to generate glucose‐mobilizing responses and activation of hepatic AC determined in the same study

Such comparisons were made in four of the studies selected from Table 2 and listed in Table 3. The concentration gaps were revealed by comparisons of TCs of two glucagon dose‐response curves: one—with a lower concentration range—for generating the indicated glucose‐mobilizing response, and the other—over a higher range—for increasing either tissue cAMP levels or AC activity. As Table 3 shows, all of the minimal concentrations required to produce glucose‐mobilizing responses were either 10 or 20 pM, but those necessary to produce AC‐activating responses were much higher, 200–1000 pM. The ratios of TCs required to activate the AC/cAMP pathway to those sufficient to activate the PLC/IP3 pathway varied from a low of 10 to a high of 100. Overall, these findings provide additional persuasive evidence that the concerns raised by Sutherland and coworkers in 1971 as discussed above—that hepatic portal plasma concentrations may be too low to activate hepatic AC—seem to be valid after all, at least for adult mammals under normal metabolic conditions. The findings summarized in Table 3 can be extended to hypothesize that all of the glucose‐mobilizing responses listed in Table 2 were produced by activation of the PLC/IP3 signal pathway without activation of AC or PKA, because all of the TCs are at or below 40 pM (Table 3).

**TABLE 3:** Differences in threshold concentrations (TCs) of glucagon required to generate glucose‐mobilizing responses versus those required to activate the AC/cAMP pathway

TCs (pM) required:		B/A	Reference
A. to generate the glucose‐mobilizing response	B. to increase AC activity or cAMP levels		
20	200	10	Exton, Lewis, et al. (1971)
10	250	25	Wakelam et al. (1986)
10	400	40	Blackmore and Exton (1986)
10	1000	100	Corvera et al. (1984)

Note

All values are from those references cited in Table 2 that reported both glucagon TCs – one to generate the indicated response and the other to activate AC or increase tissue cAMP levels—in the same study. Glucose‐mobilizing responses include the stimulation of inositol‐phosphate generation, GPase activity, and glycogenolysis. Note that all of the TCs sufficient to generate the responses (10 or 20 pM) are at the low end of zone 1 (physiological; 0–60 pM). By contrast, all of the TCs sufficient to activate AC or increase tissue levels of cAMP in these studies are in zone 3 (physiological hyperglucagonemia; 100–800 pM), or zone 4 (pharmacological; >800 pM) (Figure 2).

## 6 QUESTIONS OF SIGNAL AMPLIFICATION

Observations that glucagon can stimulate hepatic glucose mobilization at physiological concentrations without measurably increasing tissue cAMP levels cannot be explained by its activation of a latent, amplified AC/cAMP pathway that is below the limit of detection.

### 6.1 The AC/cAMP pathway

**FIGURE 6:**
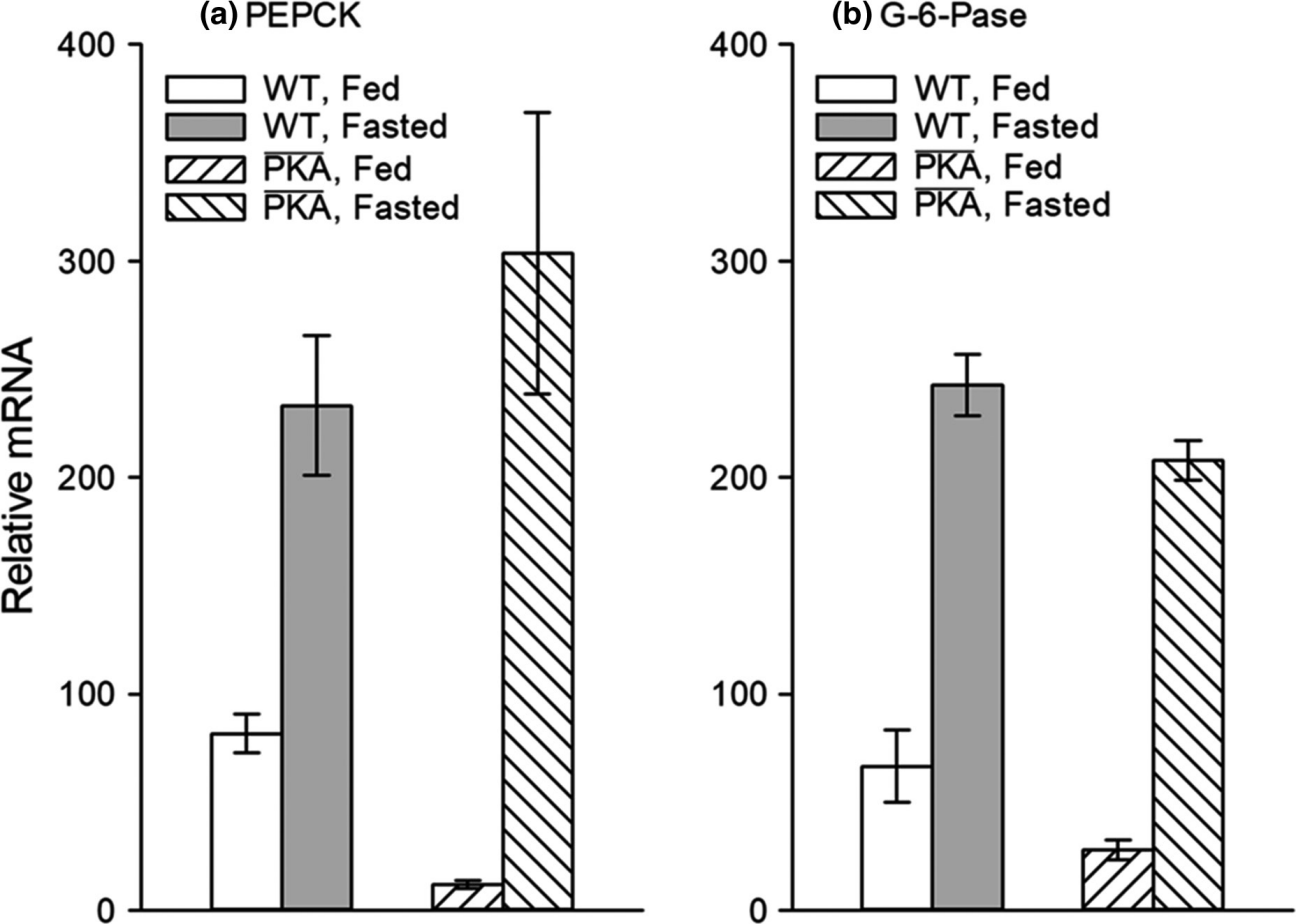
Hepatic PEPCK activity (a) and G6P activity (b) of fed or fasted (24 h) wild‐type mice (open and shaded bars) and mice with liver‐specific inhibition of PKA (cross‐hatched bars). The mutation suppressed basal cellular PKA activity by approximately 55%. The data show that substantial inhibition of hepatic PKA activity had no effect on fasting‐induced stimulation of hepatic PEPCK or G6Pase activity. The results are consistent with the hypothesis that fasting increases expressions and activities of PEPCK and G6Pase activity by activating the PLC/IP3/CaM pathway without activating the AC/cAMP pathway. However, alternative interpretations are possible because the mutation did not result in complete inhibition of PKA activity (see text). The data are from Willis et al. (2011). Note also that PKA inhibition substantially decreased constitutive (basal) expressions of PEPCK and G6Pase, suggesting that basal AC/cAMP activity is involved in regulating hepatic glucose mobilization in the absence of the activation of the pathway by glucagon.

An argument that has been occasionally put forward is that the AC/cAMP/PKA pathway in the adult liver is indeed activated by physiological plasma concentrations of glucagon, but the signal is latent, below the limit of detection by conventional methods. Recall that the consensus TC for activating AC ex vivo is 100 pM (Figure 4), but mean hepatic portal plasma glucagon concentrations are around 44 pM (Figures 1, 2b, and 4). The question is whether the AC/cAMP pathway contributes to the mediation of glucagon’s glucose‐mobilizing effects at physiological concentrations in vivo but its activation of that pathway is not detectable by conventional methodologies. A hidden role for cAMP at low glucagon concentrations had been suggested very early on by Exton and coworkers (Exton, Lewis, et al., 1971; Exton, Robison, et al., 1971): “The fact that glycogenolysis was stimulated in these experiments by concentrations of glucagon lower than those that produced a measurable elevation of tissue cyclic AMP [200 pM in their hands] suggests that the increase in nucleotide required to activate phosphorylase is extremely small, and that measurements of tissue nucleotide are a poor index of the level of metabolically active compound.” It should be borne in mind that they made that statement fifteen years prior to the publication of the discovery of the other signal (Wakelam et al., 1986). Nevertheless, because AC can be compartmentalized (Yamatani et al., 1998), PKA can be localized near the plasma membrane of the cell, cAMP phosphodiesterases can be in close proximity to the localized AC enzyme (Ong & Ambudkar, 2020; Taskén & Aandahl, 2003), and glucagon can stimulate hepatic phosphodiesterases (Heyworth & Houslay, 1983), it is theoretically possible that binding of a small fraction of the available GR2 receptors can generate enough cAMP to briefly and locally activate PKA, with amplification of the signal pathway downstream (Christophe, 1995; Jelinek et al., 1993). Responses to concentrations below 100 pM could thus be mediated by both pathways acting in concert, one overt and one latent (Figures 1, 2, 4, and 6).

Results of a key study carried out by Cherrington and coworkers (Rivera et al., 2010) seem to refute that hypothesis. The main AMPKα phosphorylation target for CaMKKβ is Thr172, and one for PKA is Ser 485 (Fujiwara et al., 2016; Hurley et al., 2006; Jacquel et al., 2018; Longuet et al., 2008; Salminen et al., 2016). Infusion of glucagon into anesthetized dogs increased plasma glucagon concentration from 14 pM to a fairly steady 57 pM (Rivera et al., 2010). At those concentrations, sufficient to increase PEPCK expression ‐ and substantially enhance glucose output ex vivo (Figure 4) ‐ glucagon increased the phosphorylation of AMPKα at Thr172, the CaMKKβ phosphorylation site, but did not enhance the phosphorylation of the enzyme at Ser485, the PKA target. These results support the central thesis of this review, that the day‐to‐day regulation of hepatic glucose mobilization by glucagon ‐ in healthy, fed and short‐term fasted experimental animals and humans – is mediated by the PLC/IP3/Ca/CaM pathway exclusively. Additional evidence against the latent signal hypothesis may have been provided by the results of a study carried out by McKnight and coworkers (Willis et al., 2011). Substantial (app. 56%) liver‐specific inhibition of PKA in mice did not diminish 24‐h fasting‐induced increases in the expressions of PEPCK, GPase (Figure 6), glyceraldehyde‐3‐phosphate dehydrogenase, the glucose transporter GLUT2, or glucose disposal. Growth and development were not different from those of the wild type. Plasma glucagon levels were not measured. These findings can be interpreted at least two ways: (1) The AC/cAMP pathway does not contribute to the regulation by glucagon of hepatic glucose mobilization in fasting mice at rest. Instead, the exclusive mediator is the PLC/IP3 pathway; or (2) The residual PKA activity may have been sufficient to at least contribute to the mediation of glucagon’s effects. Indirect evidence supports the first interpretation. Mice with liver‐specific knockout of the Gαs subunit, eliminating activation by glucagon of GR2 receptors coupled to AC but not of GR1 receptors coupled to Gαq and PLC, had normal lifespans, growth rates, and metabolic rates, increased glucose tolerance, and normal fasting glucose levels in spite of reactive hyperglucagonemia (Chen et al., 2005).

Although glucagon does not appear to activate an amplified AC/cAMP pathway, it does seem to activate an amplified PLC/IP3 signal at physiological concentrations to stimulate glucose mobilization.

### 6.2 The PLC/IP3 pathway

Although glucagon does not appear to generate an amplified AC/cAMP signal, it does seem to activate an amplified PLC/IP3 pathway at physiological concentrations. As mentioned earlier, the magnitude of the increase in IP3 in the response to physiological concentrations of glucagon is small when compared to the relatively robust increase in glucose output (Figure 4b) “Since the concentrations of IP3 produced by glucagon are significantly smaller than those produced by the ‘classical’ Ca^2+^‐mobilizing hormone vasopressin, it has been difficult to ascertain the role of a PLC‐dependent mechanism in physiological effects of glucagon” (Aromataris et al., 2006). The plausibility that activation of GR1 receptors generates an amplified signal downstream, however, is supported by histological observations. As with the AC/cAMP pathway, components of the PLC/IP3 pathway are localized and compartmentalized within or close to the cell membrane. Much of the ER membrane containing the IP3Rs is closely applied to the plasma membrane (Feriod et al., 2014; Ong & Ambudkar, 2020). The proximity of GR1 receptors and PLC in the plasma membrane to IP3Rs in the ER has been proposed to facilitate signal transfer to an extent that is sufficient to produce a substantial increase in cellular calcium and related downstream events leading to enhanced glucose output (Hansen et al., 1998; Ong & Ambudkar, 2020; Williamson et al., 1987). As discussed above, concentrations of glucagon that produce no measurable increase in tissue cAMP nonetheless produce substantial increases in glucose output (Figure 4), glycogenolysis, gluconeogenesis, and altered activities of glucose‐mobilizing enzymes (Table 2, Figure 5) that can only be attributed to activation of the PLC/IP3 pathway. Even though IP3 is only 10% of all inositol‐phosphates generated by glucagon (Williamson et al., 1987), the “… small increase in IP3 is sufficient to fully activate phosphorylase” (Keppens et al., 1993) and presumably to produce other signal‐related downstream events as well (Figure 5), leading to enhanced glucose output without activation of AC. With regard to the question of signal amplification, the major difference between the generation of IP3 and of cAMP is that, within the physiological, zone 1 glucagon concentration range, at or below 60 pM, the former is small but consistently measurable while the latter is undetectable because it is not generated above constitutive levels (Figure 4) (Table 2, Figures 4 and 5).

The regulation by glucagon of hepatic glucose mobilization in type 2 diabetes seems to be mediated by the PLC/IP3 pathway exclusively. Whether it is also mediated by the AC/cAMP pathway in type 1 diabetes or starvation defies prediction.

## 7 UNCERTAIN INVOLVEMENT OF THE AC/cAMP PATHWAY IN HEPATIC RESPONSES TO GLUCAGON: DIABETES AND STARVATION

### 7.1 Unpredictability of the AC/cAMP response to transitional concentrations of glucagon

Evidence presented above seems to establish that mean hepatic portal plasma concentrations that prevail most of the time—that is, in metabolically unchallenged experimental animals and humans at rest—are in zone 1, below 60 pM by RIA (Figures 1 and 2b). At those concentrations glucagon regulates hepatic glucose mobilization exclusively by activating the PLC/IP3 pathway without activating AC. Those plasma concentrations are consistently found in healthy or type2 diabetic experimental animals or humans. By contrast, at zone 3 concentrations, above 100 pM, glucagon predictably activates AC (Figure 4a) so that its influences on glucose mobilization are mediated by a combination of the AC/cAMP and PLC/IP3 pathways. Those supraphysiological concentrations are characteristic of early neonates or exercising adults (Figure 2d) (Figures 1, 2, and 4).

However, within the transitional zone 2 range, between 60 and 100 pM, the effect of glucagon on AC activity is unpredictable, even ex vivo. Plasma concentrations in starving or T1D experimental animals or humans can approach or be within that range (Figure 2c). There is an apparent gap between results obtained ex vivo, which seem to show that AC is activated minimally or not at all (Figure 4), and those generated in vivo in these conditions, indicating that glucagon can inconsistently and substantially activate AC and increase tissue cAMP levels even below these transitional plasma concentrations. With regard to ex‐vivo variability, recall that in the concentration‐effect curve in Figure 4, the glucagon TC of 100 pM increased cAMP levels – in perfused livers, hepatocytes, or hepatocyte membranes ‐ by a composite mean of only about 4% of the maximum response to higher concentrations, but the individual responses were somewhat variable. Of the 15 references used to calculate that 4% mean, 6 (including the original communication by Sutherland and coworkers) reported no effect (Corvera & García‐Sáinz, 1984; Exton, Robison, et al., 1971; Lynch et al., 1989; Robberecht et al., 1988; Soman & Felig, 1978; Unson et al., 1989). The other 9 (Clark & Jarrett, 1978; Dich & Gluud, 1976; Dighe et al., 1984; England et al., 1983; Hermsdorf et al., 1989; Pohl et al., 1972; Rodbell et al., 1971; Sonne et al., 1978; Yagami, 1995) reported increases in either tissue cAMP levels or AC activity between 2% and 21% of the maximum. According to at least 3 additional sources, transitional concentrations are indeed capable of significantly activating AC either ex vivo or in vivo. Glucagon at 100 pM was reported to increase cAMP levels 86%, PKA activity 62%, and glucose output 90% over basal levels in hepatocytes isolated from fed rats (Assimacopoulos‐Jeannet et al., 1977). In perfused rat livers, 60 pM glucagon increased glucose output by about 87% of the maximum produced by 1000 pM, and increased tissue cyclic AMP 3‐fold, from 0.5 to 1.5 pmol/g (Doi et al., 2001). Co‐infusion with insulin completely blocked the increase in cAMP but reduced the peak increase in glucose output by only 25%. The residual response was presumably the result of activation of the PLC/IP3 pathway. The authors apparently did not specify whether the livers were isolated from fed or fasted rats. One report is unusual with regard to the magnitude of the increase in cAMP in this concentration range (Perry et al., 2020). At the end of a 2‐h infusion of glucagon into overnight‐fasted mice, plasma glucagon levels had increased from 9 pM into zone 2, approximately 80 pM, and hepatic cAMP had risen from 300 to 1650 pmoles/g, an increase of over 5‐fold. Values at earlier time points were not reported. As mentioned above, transitional glucagon concentrations can be achieved in at least two metabolically stressful conditions, insulin‐dependent diabetes (T1D) and starvation (Figure 2c). Not surprisingly, attendant effects on hepatic cAMP or AC activity in those conditions are variable.

### 7.2 Diabetes

**FIGURE 7:**
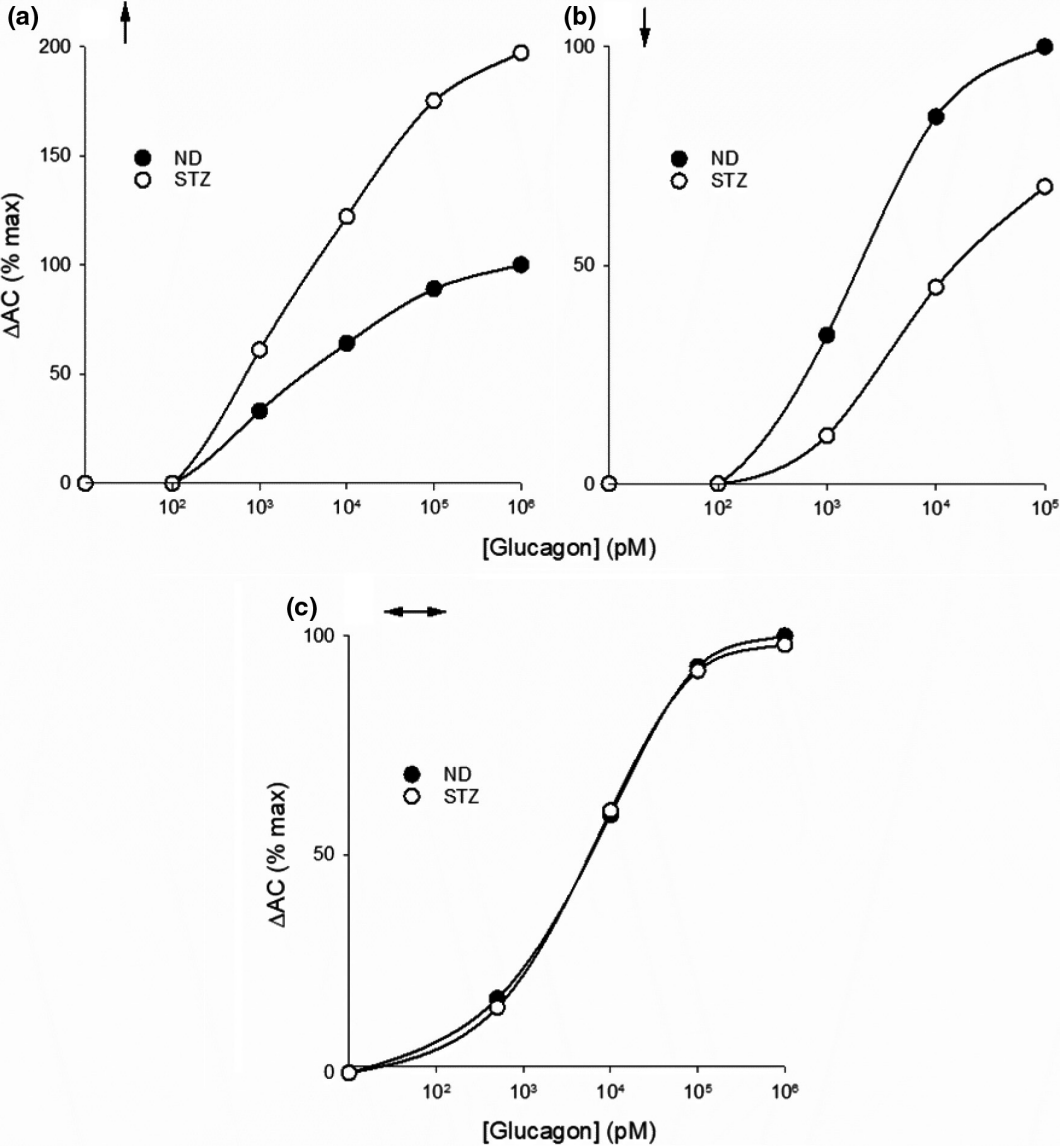
Variable effects of STZ‐induced diabetes in vivo on the concentration‐dependent stimulation of adenylate cyclase (AC) by glucagon in rat hepatocyte membrane preparations. The data are expressed as the % of the maximum produced by the nondiabetic (ND) control group. Diabetes either (a) enhanced Lynch et al. (1989); (b) suppressed Dighe et al. (1984); or (c) had no effect on Srikant et al. (1977) the activation of AC by glucagon. The doses of STZ were 100 mg/kg (a and b) or 65 mg/kg (c), and the durations of diabetes were 3 days (a) or 5 days (b and c). Note that diabetes had no effect on the sensitivity to glucagon, that is, on the TC required to activate AC (estimated to be 300 pM in a and b, and 100 pM in c), regardless of its attendant effect on the magnitude or direction of the response to increasing concentrations of glucagon but see Walsh & Dunbar, (1984). However, influences of co‐regulators such as insulin or corticosteroids, at least in STZ‐induced diabetes, may variably affect sensitivity to AC‐activating effects of glucagon in vivo (see text). For additional examples in each category, see (Allgayer et al. (1982; Soman & Felig, (1978); Walsh & Dunbar, (1984) (increased responsiveness); Chamras et al. (1980) and Yamashita et al. (1980) (decreased responsiveness); and Pilkis et al. (1974) (no effect on responsiveness). These results highlight the uncertainties with regard to the effects of T1D on the relationship between glucagon concentrations and the activation of AC/cAMP

It is becoming increasingly clear that hepatic glucose‐mobilizing effects of glucagon likely contribute to the hyperglycemia and related complications of diabetes (Foretz et al., 2005; Lee et al., 2012; Müller et al., 1973; Patil et al., 2020; Rix et al., 2019; Sharabi et al., 2019; Wewer Albrechtsen et al., 2019). Whether the elevation in blood glucose is associated with a rise in plasma glucagon seems to depend on the category of diabetes. The “hyperglucagonemia” of diabetes (Rix et al., 2019) appears to be nonexistent in either obesity with insulin resistance or T2D (hereafter referred to collectively as T2D) (Figure 2c), but it may be present at moderate levels in insulin‐dependent T1D (Figure 2c, Table 4A). As expected, hepatic AC/cAMP levels in T2D are basal and characteristic of those in nondiabetic animals, but those in T1D are variable and may exceed nondiabetic levels (Table 4A). This difference can be attributed, at least partially, to the disparity of mean plasma glucagon concentrations; normal in T2D but variably elevated in T1D. Respective effects of altered insulin action or availability may also be involved (Tables 4 and 5, Figures 2, 4, and 7).

**TABLE 4:** Plasma glucagon and hepatic AC/cAMP levels in insulin‐dependent diabetes, starvation, early neonates, and exercising adults

	Change in Plasma [glucagon]			Δ in hepatic	
	Remain in zone 1	Into zone 2	Into zone 3		
Condition	(0–60 pM)	(60–100 pM)	(100–800 pM)	AC or cAMP	Reference
A. Diabetic (vs. ND)					
Humans, Obese IR	X			↔	Livingston et al. (1985)
Rats, T2D model	X			↔	Xue, Cei, et al. (2017)
Rats, T1D	X			↑	Soman and Felig (1978)
		X		↓	Walsh & Dunbar (1984)
		X		↔	Yamashita et al. (1980)
			X	↓	Srikant et al. (1977)
B. Starvation (vs. Fed)					
Rats	X			↑	Seitz et al. (1976)
	X			↓	Srikant et al., 1977)
	X			↔	Goldstein et al. (1978)
		X		↑	Bois‐Joyeux et al. (1986)
C. Neonates (vs. Adults)					
Rats			X	↑	Beaudry et al. (1977)
D. Exercise (vs. Rest)					
Rats			X	↑	Sellers et al. (1988), Winder et al. (1981), Winder, Yang, et al. (1988) and Winder, Arogyasami, et al. (1988)
Dogs			X	↑	Issekutz (1981)

Note

**FIGURE 8:**
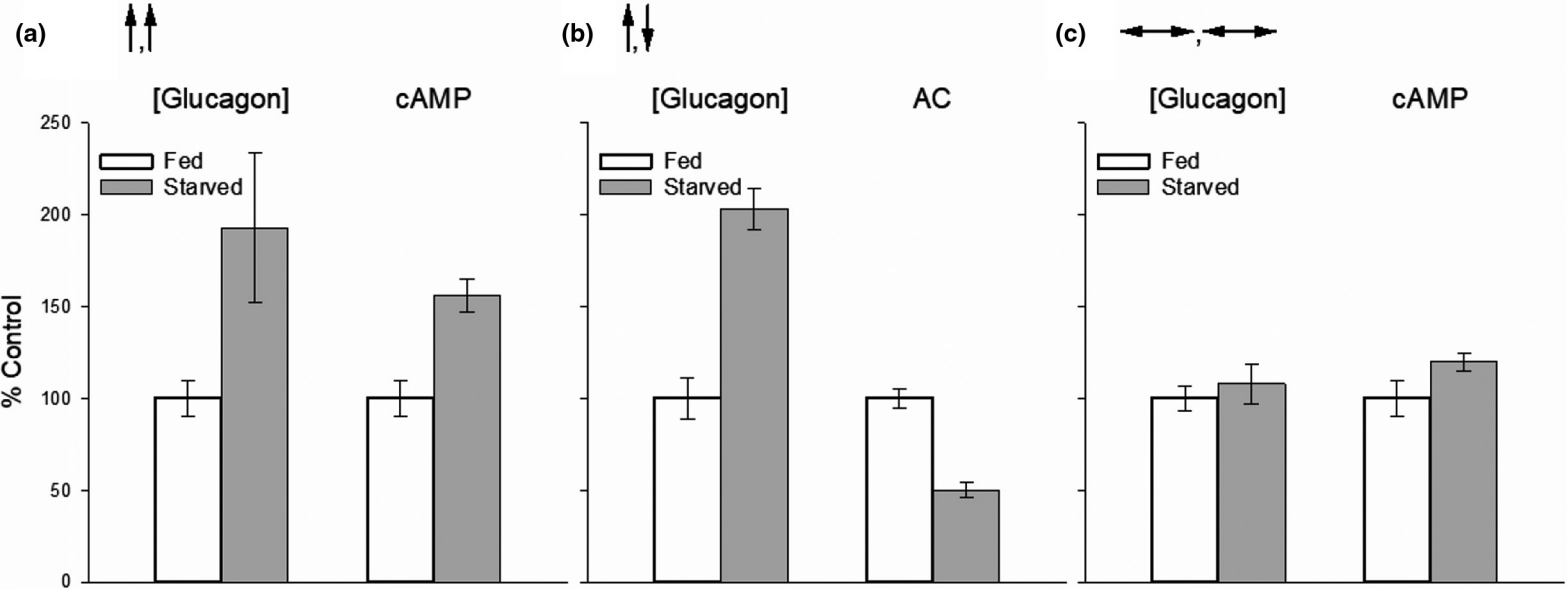
Variable effects of starvation on plasma glucagon and hepatic tissue cAMP levels or AC activity in rats. Depending on the study (see Table 4B), starvation was reported to increase both plasma glucagon and tissue cAMP levels (a), increase plasma glucagon but decrease hepatic AC activity (b), or have no effect on either plasma glucagon or tissue cAMP levels (c). The peak plasma glucagon levels in A and B are 56 and 57 pM, within zone 1 (Figure 2). The durations of starvation were 2 days (a), 6 days (b) and 3 days (c). The results highlight the uncertainty regarding the effect of starvation on the relationship between plasma glucagon and hepatic tissue cAMP levels or AC activity. Adapted from Seitz et al. (1976) (a), Srikant et al. (1977) (b), and Goldstein et al. (1978b) (c)

Listed are effects of the indicated condition on plasma glucagon and hepatic AC/cAMP levels determined in the same study. Experimental preparations were hepatocyte membranes (basal AC activity), hepatocytes, liver slices, or perfused livers (basal AC activity or tissue cAMP levels). In STZ‐induced diabetes or starvation (A and B), both plasma glucagon and associated AC activities or cAMP levels are variable (see Figures 7 and 8). By contrast, in the early neonates and exercising adults, plasma glucagon levels are consistently in zone 3 and AC/cAMP levels are predictably and consistently elevated. Mean plasma glucagon concentrations (pM) in the control groups were: nondiabetic (A), 16, 8, 18, 33, 28, and 54; fed (B), 17, 28, 38, and 46; adults (C), 36; and pre‐exercise (D), 52, 49, 66, 75, and 69. The composite mean for all control groups is 39.6 ± 5.1 pM (*n* = 16). The doses of STZ were 60–70 mg/kg, the durations of diabetes were 5–14 days, and the durations of starvation were 24–72 h.

**TABLE 5:** Variable effects of diabetes in vivo on basal hepatic AC activity or cAMP levels ex vivo

Direction of change			
in basal AC/cAMP	% of nondiabetic levels	*n*	References
Increase	346 ± 58	7	Cánepa et al. (1985), Cánepa et al. (1990), Jefferson et al. (1968), Miller et al. (1979), Pilkis et al. (1974), Soman and Felig (1978) and Walsh and Dunbar (1984)
Decrease	52 ± 4	5	Dighe et al., 1984; Doi et al., 2001; Gawler et al., 1987; Portha et al., 1983; Srikant et al., 1977)
Little to no effect	104 ± 6	5	Allgayer et al. (1982), Chamras et al. (1980), Lynch et al. (1989), Sumi et al. (1984) and Yamashita et al. (1980)
Mean ±SEM	180 ± 38	17	

Note

Values, expressed as the ratio (%) of diabetic/nondiabetic levels, are means ±SEM of basal AC activities or cAMP levels in perfused livers, hepatocytes, or liver cell membranes, calculated from combined data obtained from the indicated number of sources in each category. 15 of the studies were of the effects of STZ‐ or alloxan‐induced diabetes in rats, and 1 in mice (Sumi et al., 1984). One group reported in the same study that STZ‐diabetes had little effect (small decrease), but BB/WOR genetic diabetes increased, basal AC activity (Lynch et al., 1989). Two of the studies were of experimental models of type 2 diabetes; one induced by STZ given to neonatal rats (Portha et al., 1983) and the other of spontaneously diabetic Goto‐Kakizaki rats (Doi et al., 2001). Effects of diabetes on basal AC/cAMP levels were not well correlated with the subsequent responsiveness to administered glucagon (Figure 7). The variability could not be easily explained by differences in the dose of STZ or alloxan (50–150 mg/kg) (but see Dighe et al., 1984), duration of diabetes (2–5 days), effects on plasma glucagon levels (Chamras et al., 1980; Portha et al., 1983; Soman & Felig, 1978; Srikant et al., 1977; Walsh & Dunbar, 1984; Yamashita et al., 1980; Table 4) or changes in glucagon receptor affinities or densities (Chamras et al., 1980; Dighe et al., 1984; Srikant et al., 1977; Walsh & Dunbar, 1984; Yamashita et al., 1980).

Declines in either plasma insulin or the sensitivity to insulin in diabetes would be expected to lift its inhibition of cAMP‐mediated responses to glucagon (Gabbay & Lardy, 1984; Illiano & Cuatrecasas, 1972). Sites and mechanisms of inhibitory actions of insulin include suppressed glucagon secretion from alpha cells (Ravier & Rutter, 2005) and, in the liver, reduced basal and glucagon‐stimulated AC/PKA activities (Claus et al., 1979; Heyworth & Houslay, 1983a; Seitz et al., 1977) and accelerated cAMP hydrolysis by stimulation of cAMP‐dependent phosphodiesterase (Heyworth et al., 1983b; House et al., 1972; Houslay, 1990; Loten et al., 1978). Treatment with anti‐insulin antibodies in vivo increased basal cAMP of perfused livers ex vivo (Jefferson et al., 1968). Opposing nuclear effects of insulin include inhibition of the expressions or activities of PGC‐1α, CREB, and PEPCK (Christ et al., 1990; Hatting et al., 2018; Herzig et al., 2001). In theory, these nuclear anti‐glucagon effects of insulin would apply to either of the glucagon‐activated signal pathways. However, insulin resistance in T2D and its deficiency in T1D seem to have differing effects on plasma glucagon levels and their relationships to hepatic AC/cAMP activity.

In T2D, both plasma glucagon concentrations and basal hepatic AC/cAMP levels are within the normal range. Mean plasma glucagon levels in nondiabetic and T2D humans are similar and well within zone 1 (Figure 2a and c). According to the sources cited here, the mean peripheral venous plasma glucagon concentrations in nondiabetic and T2D humans are statistically identical, 29 and 28 pM respectively (Figure 2a and c), well within zone 1. Measured by ELISA they are even lower (Table 1). Mean plasma concentrations in normal subjects and T2D patients, measured by ELISA, were 6.0 and 9.5 pM, respectively (Kobayashi et al., 2020). Livingston et al. (1985) reported that basal, glucagon‐activated, and NaF‐stimulated AC activities in membrane preparations from liver biopsies taken from obese patients were not significantly different from those of lean patients. In both groups, plasma glucagon concentrations were below 30 pM. In a genetic model of T2D in the rat, the increase in hepatic cAMP produced by glucagon infusions in situ was actually blunted compared to the responses of nondiabetic controls (Doi et al., 2001). Similarly, glucagon‐AC dose‐response curves generated in hepatocytes or hepatocyte membranes from T2D model rats (low dose of STZ given to neonates) were depressed relative to those of the nondiabetic controls. Constitutive hepatic cAMP levels were lower in the diabetics, but glucagon receptor binding affinities and capacities and cAMP‐phosphodiesterase activities were normal (Portha et al., 1983). In a diet‐induced insulin‐resistant rat model (Xue, Wei, et al., 2017), the animals were hyperglycemic and displayed elevated expressions of PEPCK and G6Pase but normal plasma glucagon levels and basal hepatic tissue cAMP levels. Expressions of Gαs and beta adrenoceptors were elevated, but glucagon receptor expressions or densities were not reported. Evidently, in most studies of T2D plasma glucagon concentrations are normal and hepatic AC/cAMP is basal, and effects of glucagon on AC/cAMP are either normal or inhibited. It can thus be plausibly hypothesized that, because concentrations of circulating glucagon in T2D remain within zone 1 (Figure 2c), glucagon’s contribution to elevations in hepatic glucose output, and thus to the hyperglycemia, in T2D would be mediated exclusively by the PLC/IP3 pathway in vivo. But more extensive investigations will be required to confirm that hypothesis.

By contrast, the relationship between plasma glucagon concentration, hepatic glucose output, and the AC/cAMP pathway in T1D is much less clear. The contribution to the hyperglycemia attributable to glucagon‐induced increases in hepatic glucose output may be mediated by GR1 activation alone or by the activation of both GR1 an GR2 receptors, depending at least in part on the magnitude of the increase in plasma glucagon concentrations. In experimental STZ‐ or alloxan‐induced T1D, mean plasma glucagon concentrations may be normal (remain in zone 1), but may occasionally enter zone 2; that is, T1D animals may be normo‐ or moderately hyper‐glucagonemic (Table 4A). According to the sources cited in Figure 2 for T1D, peripheral venous plasma glucagon concentrations in STZ‐ or alloxan‐induced diabetes in rodents vary between a low of 20 pM and a high of 246 pM, with a mean inside the transitional zone, 78.2 ± 17.3 pM (Figure 2c). Based on the hepatic portal/peripheral venous concentration ratio of 1.5, as discussed above, the mean hepatic portal concentration in T1D, measured by RIA, would be over 100 pM. However, regardless of whether the plasma concentrations in T1D are in zones 1, 2, or 3, hepatic AC/cAMP levels can be elevated, reduced, or normal, with no apparent relationship to plasma concentrations (Table 4A).

Results of ex vivo studies are also inconsistent. Prior imposition of T1D in vivo can increase, suppress, or have no effect on glucagon‐stimulated AC activity in hepatic preparations ex vivo (Tables 4A and 5, Figure 7). Plasma glucagon levels and hepatic AC/cAMP activities may vary together or in different directions. Insulin‐dependent diabetes can produce either a moderate increase in plasma glucagon levels (i.e., fail to increase them out of zone 1) or increase them into zone 2, yet at the same time either increase or have no effect on basal AC/cAMP. It can also increase plasma glucagon all the way into to zone 3 and actually *decrease* basal AC/cAMP (Table 4A). In three conflicting studies (Figure 7), T1D decreased basal AC activity and the AC‐activating responsiveness to increasing concentrations of glucagon (Dighe et al., 1984), had no effect on basal activity but increased the responsiveness to glucagon (Lynch et al., 1989), or decreased basal activity but had no effect on responsiveness to glucagon (Srikant et al., 1977). In the first two studies (Dighe et al., 1984; Lynch et al., 1985), the effects of diabetes on plasma glucagon levels were not reported. But in the third (Srikant et al., 1977), the suppressed basal AC activity was observed ex vivo after diabetes had produced a marked increase in plasma glucagon levels in vivo (103 pM from 28 pM). Reduced basal AC activities were associated with lower densities and affinities of the GR2 receptor (Dighe et al., 1984; Srikant et al., 1977). But basal AC activities and receptor densities are not always predictive of subsequent responsiveness to glucagon. The responses may be suppressed (Dighe et al., 1984) or unaffected (Srikant et al., 1977). Furthermore, when STZ‐induced diabetes does alter the position of the glucagon‐AC/cAMP dose response curve in hepatocyte preparations—up or down—it displaces it vertically (i.e., it alters glucagon’s effectiveness) and not horizontally (i.e., it does not alter its potency) (Figure 7) (but see Walsh & Dunbar, 1984). Thus, the TC ex vivo for activating (or inhibiting) AC in diabetes seems to be at least 100 pM, as it is in hepatocyte preparations isolated from nondiabetic animals. By extension, when T1D does not increase plasma glucagon above 60 pM, in vivo it should not alter hepatic cAMP levels either. Yet apparently it can (Soman & Felig, 1978 in Table 4A). None of these inconsistencies is obviously related to the sex of the animals, dose of STZ or alloxan, or duration of diabetes (Dighe et al., 1984; Srikant et al., 1977; Walsh & Dunbar, 1984).

Studies are also conflicting with regard to the contribution of glucagon‐induced stimulation of hepatic glucose output to the hyperglycemia of T1D. Treatment with STZ, sufficient to produce profound hyperglycemia in wild‐type mice, had no effect on glycemia in glucagon receptor knockout mice (Lee et al., 2012). Results of control studies led the authors to conclude that elimination of hepatic glucose‐mobilizing actions of glucagon also eliminates diabetic hyperglycemia, and that hyperglycemic actions of glucagon are more important than those of insulin withdrawal or sub‐sensitivity in the pathogenesis of the disease. They did not discuss, however, whether the ameliorative effects of glucagon receptor knockout in their hands were the result of elimination of GR2 receptors coupled to AC alone or of both GR2 and GR1 receptors coupled to PLC. In apparent conflict with those findings, administration of the glucagon‐like peptide‐1 (GLP‐1) agonist (and inhibitor of glucagon secretion) liraglutide to STZ‐diabetic rats normalized the increase in plasma glucagon without significantly affecting the hyperglycemia (Meek et al., 2015). The authors interpreted those results as evidence that the hyperglycemia of T1D is not dependent on the rise in plasma glucagon. But the increase in plasma glucagon in the diabetic animals that they observed was moderate, 21.5 pM in T1D vs. 14.4 pM in the nondiabetic controls, sufficient to increase glucose output only to 10% of the maximum according to the dose‐response curve in Figure 4, and in any case well within zone 1. Thus, the stimulated output was presumably mediated exclusively by increased GR1 receptor activation. Anti‐glucagon antibodies bound to and neutralized all of the circulating glucagon and consequently decreased blood glucose in nondiabetic controls by about 33% in nondiabetic controls and 40% in alloxan‐diabetic, hyperglycemic rabbits (Brand et al., 1996). Thus, neither the relative contribution of glucagon‐induced hepatic glucose output, nor those of GR‐1 and GR‐1 receptor activation, to the hyperglycemia of T1D is clearly defined. Apparently, potential hypoglycemic actions of the PLC inhibitor U73122, in the absence or presence of T1D, have not been investigated. One reason for the presumed hesitancy could be that the results would be difficult to interpret because U73122 has nonspecific effects in vivo (Bill & Vines, 2020) including interference with insulin‐induced increases in skeletal muscle glucose uptake (Wright et al., 2002).

To summarize, in T2D plasma glucagon levels are in the normal range, and thus would not be expected to activate hepatic AC in vivo. Any glucagon‐induced increases in hepatic glucose output in that condition would presumably be mediated by the PLC/IP3 pathway exclusively. In T1D plasma glucagon concentrations are quite variable, ranging between normal and hyperglucagonemic. Further, there is no obvious correlation between plasma concentrations and the activation of hepatic AC. Thus, the role of the AC/cAMP pathway as a co‐mediator, with the PLC/IP3 pathway, of the hepatic glucose‐mobilizing and hyperglycemic actions of glucagon in T1D remains unclear.

### 7.3 Starvation

Starvation (defined here as fasting for 24 h or more) has complex direct and indirect effects on plasma glucagon, glycemia, and hepatic glucose metabolism. Depending on the duration, plasma glucose levels may be fairly well maintained or may fall somewhat as starvation progresses (Blommaart et al., 1995; Mlekusch et al., 1981; Smadja et al., 1990), and plasma corticosterone concentrations can remain fairly steady or increase (Mlekusch et al., 1981; Ogias et al., 2010). Expressions of PEPCK and G6Pase rise (Krone et al., 1976; McNeill et al., 1982; Mlekusch et al., 1981; Ogias et al., 2010; Seitz et al., 1977), while plasma insulin‐to‐glucagon ratios generally decline (Aguilar‐Parada et al., 1969; Bois‐Joyeux et al., 1986; Goldstein et al., 1978; Mlekusch et al., 1981; Shiota et al., 1996; Smadja et al., 1990; Verrillo et al., 1988). Increases in PEPCK expression and gluconeogenesis during starvation are mediated in part by declines in hepatic levels of G protein receptor‐coupled kinase 2 (GRK2) (Cruces‐Sande Arcones et al., 2020). Decreases in GRK2 inhibit the internalization of G proteins associated with glucagon receptors (and other GPCRs), preserving their responses to starvation‐induced increases in plasma glucagon concentrations (when they occur) (Table 4, Figures 2, 6, and 8).

As in experimental T1D, in starvation the relationship between plasma glucagon and hepatic AC/cAMP is variable and difficult to predict. According to the references cited here (Figure 2c), the collective mean peripheral venous plasma glucagon concentration in starvation of rats or humans is 48 pM, approaching zone 2. All of the measurements used to generate that mean were taken by RIA from the peripheral venous circulation. Applying the hepatic portal/peripheral venous ratio of 1.5 (Figure 2b), the corrected concentration in the hepatic portal circulation would be 72 pM, well into the transitional zone 2. The mean values, measured in the peripheral venous circulation, varied from a low of 34 to a high of 66 pM. Even when corrected, none enters zone 3 (exceeds 100 pM) and thus would not be expected to increase hepatic AC activity or cAMP levels. However, hepatic AC activity or cAMP levels may increase anyway (Seitz et al., 1976), but they may also decrease (Srikant et al., 1977), or remain unaltered (Goldstein et al., 1978), with no obvious relationship to attendant changes in plasma glucagon levels (Table 4B, Figure 8). In one representative report, starving for 24 h slightly decreased plasma glucagon in rats from 57 ± 4 to 50 ± 11 pM, but *increased* hepatic tissue cAMP by over 60%, from 0.16 ± 0.01 to 0.26 ± 0.02 nmoles/g wet wt. (Goldstein and Curnow (1978)). After starving for 120 h, plasma glucagon concentrations rose to 107 pM, and hepatic cAMP levels further increased to 0.40 nmoles/g. In contrast, rats starved for 6 days exhibited increased plasma glucagon from 28 to 57 pM, but *reduced* hepatic AC activity from 1.27 to 0.64 nmoles/10 min. · mg prot.^−1^ (Srikant et al., 1977). As mentioned above, Willis et al. (2011) reported that liver‐specific inhibition of PKA (by about 60%) in adult mice had little or no effect on hepatic responses to starving for 24 h (Figure 6). It did not suppress starvation‐induced increases in hepatic PEPCK or G6Pase expression or changes in blood glucose levels. In the fed and starved states, both PKA‐inhibited and wild‐type mice had similar or identical expressions of hepatic glucokinase, G6Pase, and GLUT‐2 transporters, and increases in the starvation‐induced expression of GAPDH, hepatic glucose disposal, body weights, liver weights, and blood insulin levels. As discussed above, the results can be interpreted at least two ways: (1) The effects of starvation do not involve activation of the AC/cAMP pathway; or (2) The residual basal PKA activity was sufficient to mediate or at least contribute to the responses. The authors did not measure plasma glucagon levels, but it seems likely that they were within zone 1, below 60 pM, in the fed controls (Figures 1 and 2a). Interestingly, PKA inhibition did significantly suppress expressions of PEPCK and G6Pase in that group. A possible implication of those findings is that the expressions of PEPCK and G6Pase are maintained at minimal levels at least in part by a steady, constitutive, unstimulated AC/cAMP/PKA signal at plasma glucagon levels below at least 60 pM. Expressions of these enzymes above the basal level would then be controlled by variations in plasma glucagon concentrations within that range, mediated by the PLC/IP3/PLC pathway exclusively. To carry that speculation to an extreme, this principle might apply to all intracellular targets—shared by the two pathways—that are involved in the regulation of hepatic glucose mobilization. In starvation, therefore, as in T1D, neither the relationship between plasma glucagon concentrations and hepatic tissue cAMP levels nor the role of the AC/cAMP signal pathway in mediating glucagon’s glucose‐mobilizing effects is clearly established. Glucagon’s control of hepatic glucose mobilization in these conditions may be mediated by the PLC/IP3 pathway alone or in combination with the AC/cAMP pathway.

In contrast to its variable effects in type 1 diabetes and starvation, in early neonates or exercising adults glucagon consistently activates the hepatic AC/cAMP pathway. Thus, the hormone adaptively boosts hepatic glucose output in order to meet the elevated systemic demand for glucose in both of these metabolically stressful conditions.

## 8 ROLE OF THE AC/cAMP PATHWAY IN MEETING ELEVATED GLUCOSE DEMAND IN EARLY NEONATES AND EXERCISING ADULTS

As discussed above, when systemic glucose supply/demand ratios fall to critical levels, plasma glucagon concentrations consistently rise from zone 1 into zone 3 (physiological hyperglucagonemia) and thus predictably activate both the AC/cAMP and PLC/IP3 pathways simultaneously. As the ex vivo data presented in Figure 4 predict, at those higher concentrations activation of the AC/cAMP pathway serves as a backup signal in vivo, supplementing a PLC/IP3 pathway that is also further stimulated, boosting hepatic glucose mobilization to meet the elevated systemic glucose demand. This pattern is consistently found in two metabolically stressful conditions, the early neonate and the exercising adult.

### 8.1 Early neonates

**FIGURE 9:**
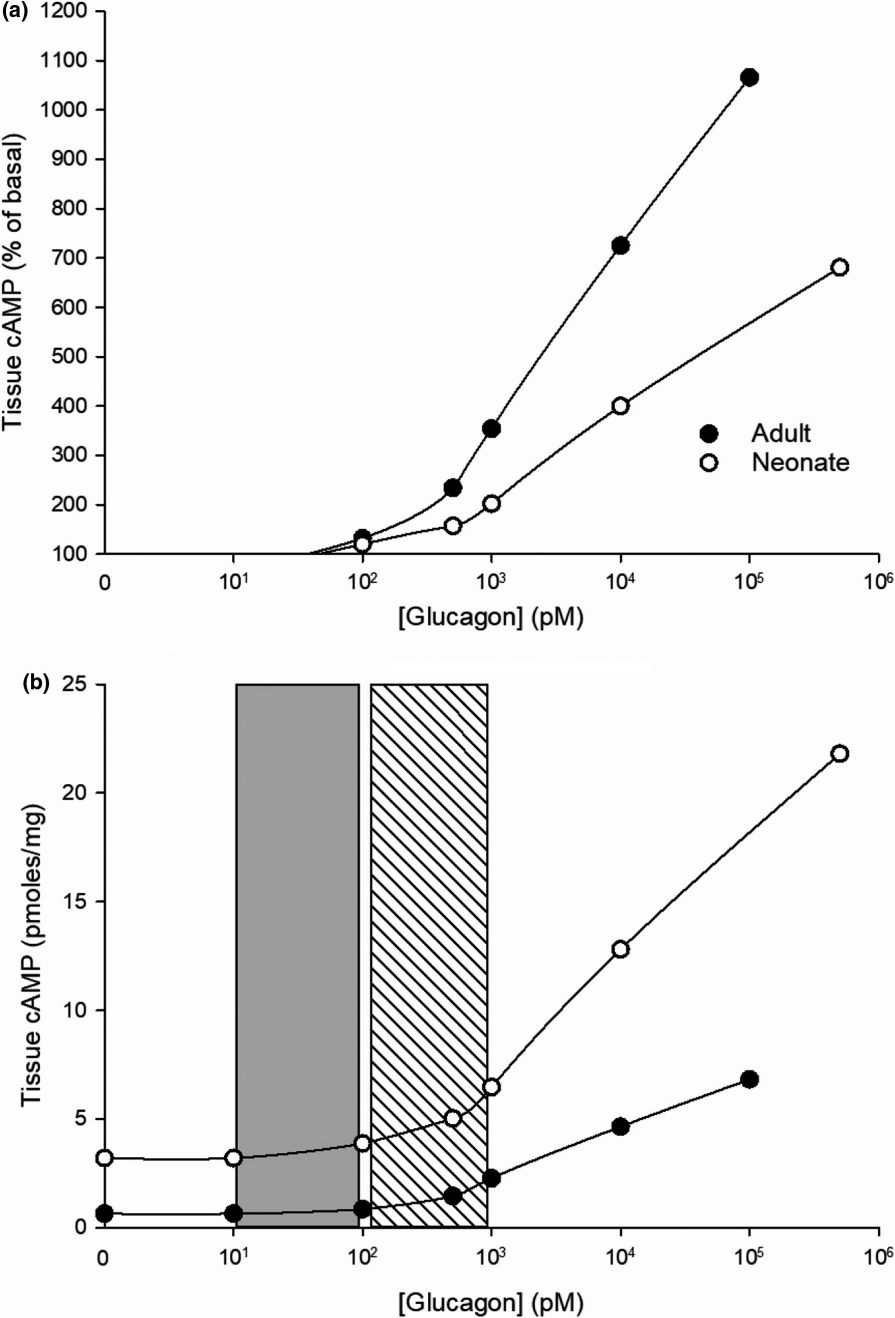
Comparison of the concentration‐dependent effects of glucagon on cAMP levels in neonatal and adult rat hepatocytes, adapted from Beaudry et al. (1977). (a) The data are expressed as reported, in relative terms (% of basal). (b) The same data when converted here to absolute levels (pmoles/mg wet wt.). The shaded bar depicts the full hepatic portal glucagon concentration in the adult from Figure 1, and the crosshatched bar corresponds to the range of peripheral venous plasma glucagon concentrations from just after birth to 16 h after birth (Girard et al., 1973; Sperling et al., 1974). The latter was adjusted to estimate the hepatic portal plasma concentration range according to the hepatic portal/peripheral venous ratio of 1.49 as depicted in Figure 2B. Note that the responsiveness to glucagon appears to be depressed in the neonate when expressed in relative terms (A), but that both basal and glucagon‐stimulated cAMP levels are revealed to be elevated in neonatal hepatocytes when expressed in absolute terms (B). The results in B are consistent with the view that both the constitutive and glucagon‐stimulated AC/cAMP pathway are adaptively enhanced in the neonate (see text)

Neonates are at high risk of hypoglycemia, and are uniquely dependent on the nutritional provision of exogenous glucose or, failing that, of gluconeogenic substrates, to maintain glycemia (Decaux et al., 1986; Girard, 1986; Girard et al., 1973; Mehta et al., 1987; Stanley et al., 2015). In the early neonate, moderate hyperglycemia minimizes the risk of hypoglycemic damage, particularly to the developing central nervous system (Güemes et al., 2016; Hume et al., 2002). Whole‐body knockout of cytosolic PEPCK, for example, is lethal in the early postnatal period (Semakova et al., 2017). Prematurely‐born infants appear to have abnormally low peak plasma glucagon levels, a blunted glucagon response to variable nutritional conditions (Bak et al., 2014; Hawdon et al., 1993; Hawdon et al., 1993; Hume et al., 2002; Jackson et al., 1997; Mehta et al., 1987; Molinari et al., 1982; Sunehag et al., 1994), and suppressed responses to exogenous glucagon (Hume et al., 2002). This helps to explain why premature infants are more vulnerable to damaging effects of fasting or restricted nutrient availability than are full‐term neonates (Table 4C, Figures 2d and 9a).

Metabolic and hormonal changes begin prior to birth in anticipation of the elevated glucose demand in the early neonatal period. In utero, glucose availability is sufficient in the maternal/fetal circulation, as long as maternal glycemia is not compromised. Prior to the abrupt transition from maternal to autonomous circulation at birth, hepatic glycogen stores begin to increase (Mayor & Cuezva, 1985). Relative alpha cell mass (μg/g BW) also begins to expand in utero, and can reach levels in 4‐day‐old neonates nearly 20 times greater than that of the adult (Fernández‐Milán et al., 2013; Movassat et al., 1997). In rats and humans, plasma glucagon spikes within the first hours after birth (Aalinkeel et al., 1999; Fernández‐Milán et al., 2013; Hahn et al., 1977; Lyonnet et al., 1988; Ogata et al., 1987, 1988), with mean levels that can exceed 500 pM (Girard, 1990; Luyckx et al., 1972) (Figure 2d). Hepatic gluconeogenesis does not become active until shortly after birth (Schaub et al., 1972) and increases substantially and rapidly to levels above those in the adult liver (Beaudry et al., 1977). The high insulin‐to‐glucagon ratio in utero is quickly and substantially reversed in the early neonate (Fernández‐Milán et al., 2013; Girard, 1990; Hahn & Hassanali, 1982; Ktorza et al., 1985; Lyonnet et al., 1988). A reduced counterregulatory influence of insulin on hepatic glucose handling, potentially affecting either glucagon‐activated pathway, is an important hormonal component of the adaptive response in the neonate (Girard, 1990; Hahn & Hassanali, 1982; Mlekusch et al., 1981; Nurjhan et al., 1985). A rise in plasma corticosteroid levels contributes to the enhancement of gluconeogenic gene expression (Ogias et al., 2010). Further, the rate of hepatic glucagon degradation is much lower in the fetus and early neonate than it is in the adult (Vinicor et al., 1976), presumably increasing local concentrations in the hepatic circulation and prolonging and intensifying the glucose‐mobilizing effect.

A supplementary capacity for hepatic glucose mobilization contributes to meeting the higher systemic glucose demand in the neonate compared with that in the adult (Hahn et al., 1977; Hahn & Hassanali, 1982; Ktorza et al., 1985; Mayor & Cuezva, 1985). Glycogen stores, built up in utero, are rapidly depleted postnatally, particularly during periods of fasting (Biondi & Viola‐Magni, 1977; Nurjhan et al., 1985). Compared to measurements taken from the adult, in the neonate plasma glucagon, corticosterone, and catecholamines are elevated and insulin levels much lower, while the activities and expressions of PEPCK, G6Pase, PK, and F‐1,6‐DP are all elevated (Baly et al., 1985; Beaudry et al., 1977; Biondi & Viola‐Magni, 1977; Girard, 1986, 1990; Lyonnet et al., 1988; Mayor & Cuezva, 1985; Noguchi et al., 1985; Ogata et al., 1987, 1988; Slotkin et al., 1995). Starvation in 3‐week‐old neonates for 24 h further increases plasma glucagon and PEPCK activity, depletes hepatic glycogen, and decreases plasma glucose and plasma insulin‐to‐glucagon ratios (Claeyssens et al., 1992).

Predictably, the hepatic AC/cAMP pathway is more active and responsive to glucagon in the neonate than it is in the unstressed adult. Both basal and glucagon‐activated hepatic AC activity (Beaudry et al., 1977; Girard, 1990; Slotkin et al., 1996; Vinicor et al., 1976) are markedly elevated in the neonatal liver compared to those of the adult (Table 4C). These findings are consistent with the observations that glucagon receptor densities (Pingoud et al., 1982) and the Km and V_max_ of AC are higher than they are in the adult (Vinicor et al., 1976). As the animal ages and chronic systemic demand for glucose progressively declines in the fed or short‐term fasting state, relative alpha cell mass, plasma glucagon, insulin, and insulin‐to‐glucagon ratios, corticosteroid and catecholamine concentrations, hepatic constitutive AC activity and cAMP levels, and basal rates of glucose output, steadily approach adult levels. The evidence therefore strongly supports the view that, in the early neonatal period, physiological hyperglucagonemia (Figure 2d) strongly and consistently activates the AC/cAMP pathway, enhancing activities and expressions of gluconeogenic enzymes and hepatic glucose output above adult levels in order to meet the elevated systemic glucose demand. The relative contribution of the PLC/IP3 pathway to the regulation of glucose mobilization in the neonatal liver has apparently not been investigated. It would likely be strongly activated, based on the glucagon‐inositol‐phosphate curve for adults shown in Figure 4.

In contrast with the findings discussed above, it has been reported that the activation of the AC/cAMP pathway by glucagon is paradoxically depressed in the neonate (Beaudry et al., 1977). But that interpretation may depend on how the data are presented. When expressed in relative terms (% of basal levels that are normalized to 100%) as originally reported, glucagon‐stimulated cAMP levels appear to be lower in neonatal rat hepatocytes than they are in adult hepatocytes (Figure 9a). However, when the same data are instead converted to absolute tissue concentrations (pmoles/mg), as they have been here (Figure 9b), both basal and glucagon‐stimulated cAMP levels are revealed to be higher in neonatal hepatocytes than they are in adult cells, consistent with the findings discussed above. Predictably, the elevated basal cAMP levels in the hepatocytes were evident over the glucagon concentration range corresponding to physiological hepatic portal plasma levels shown in Figure 1, while the enhanced glucagon‐simulated cAMP levels were observed at higher concentrations representative of those in neonatal plasma. The relative response to higher glucagon concentrations is lower because the basal levels are higher. It should be acknowledged, however, that neither the higher basal cAMP levels nor the increased AC/cAMP responsiveness to glucagon in the neonate, even when expressed in absolute terms, is consistently reported (Pingoud et al., 1982; Vinicor et al., 1976). The disparate results remain to be resolved.

### 8.2 Exercising adults

As in the early neonate, in adult mammals undergoing strenuous exercise metabolic demand for glucose is elevated. Acute hormonal responses to exercise recapitulate the neonatal situation at least four ways: higher plasma glucagon; increased hepatic cAMP levels; elevated plasma corticosteroid levels; and diminished plasma insulin‐to‐glucagon molar ratios (Banzet et al., 2009; Watanabe et al., 1991; Winder, 1988; Winder et al., 1988). As they do in the neonate, all four work in concert to increase hepatic glucose output in the exercising adult (Table 4D and Figure 2d).

Strenuous exercise, especially when combined with fasting or starvation, seems to be a powerful stimulus for recruitment of the AC/cAMP pathway to adaptively boost hepatic glucose mobilization (Table 4D). Exercise, alone or in combination with starvation (Winder et al., 1979, 1982), can increase mean plasma glucagon concentrations into zones 2 or 3. According to the 9 sources listed in Figure 2d, the collective mean is 236 ± 83 pM with a range of 32 to 717 pM. When plasma glucagon levels in exercise rise above 100 pM, the AC/cAMP pathway is consistently activated (Table 4D). This is associated, in both exercise and fasting, with increased hepatocyte glucagon receptor densities, but with increased receptor affinities only in response to fasting (Mehta et al., 1987; Melanς). After 20 min of exercise, both fed and overnight‐fasted rats displayed depleted hepatic glycogen levels (Winder et al., 1979). Exercise of rats to exhaustion increased the hepatic expression of PEPCK 4‐fold (Banzet et al., 2009). Strenuous exercise increased plasma glucagon from 66 to 124 pM, reduced plasma insulin from 290 to 90 pM, and increased hepatic tissue cAMP levels from 0.73 to 1.36 pmoles/mg (Winder, Yang, et al., 1988). Rats subjected to a 24‐h period of starvation and an uphill treadmill exercise for 60 min displayed a four‐fold rise in plasma glucagon (from 40 to 167 pM), a 50% drop in plasma insulin, an increase in liver cAMP of 54%, a decline in blood glucose of 55%, a ten‐fold increase in plasma corticosteroids, and nearly 100% depletion of liver glycogen (Winder et al., 1982). After 20 min of exercise, plasma glucagon was elevated and hepatic glycogen was depleted in both fed and overnight‐fasted rats, but hepatic cAMP was elevated only in the fasted animals (Winder et al., 1979). In retrospect, the cAMP‐independent action of glucagon to deplete hepatic glycogen in the exercising fasted animals can be explained by activation of the PLC/IP3 pathway alone.

In summary, the metabolic adjustments in the exercising adult recapitulate those in the early neonate in at least two important ways: (1) Plasma glucagon levels markedly increase; and (2) The higher levels of glucagon consistently activate the AC/cAMP pathway to boost hepatic glucose output in order to adaptively meet the elevated systemic glucose demand. In this regard, the role of the AC/cAMP pathway in mediating the effects of glucagon is much clearer than it is in T1D or starvation alone.

Glucocorticoids may inconsistently enhance the ability of glucagon to increase hepatic tissue levels of cAMP by increasing glucagon’s effectiveness but not its potency in activating AC.

## 9 POSSIBLE ROLE OF ELEVATED GLUCOCORTICOIDS IN STARVATION AND DIABETES

**FIGURE 10:**
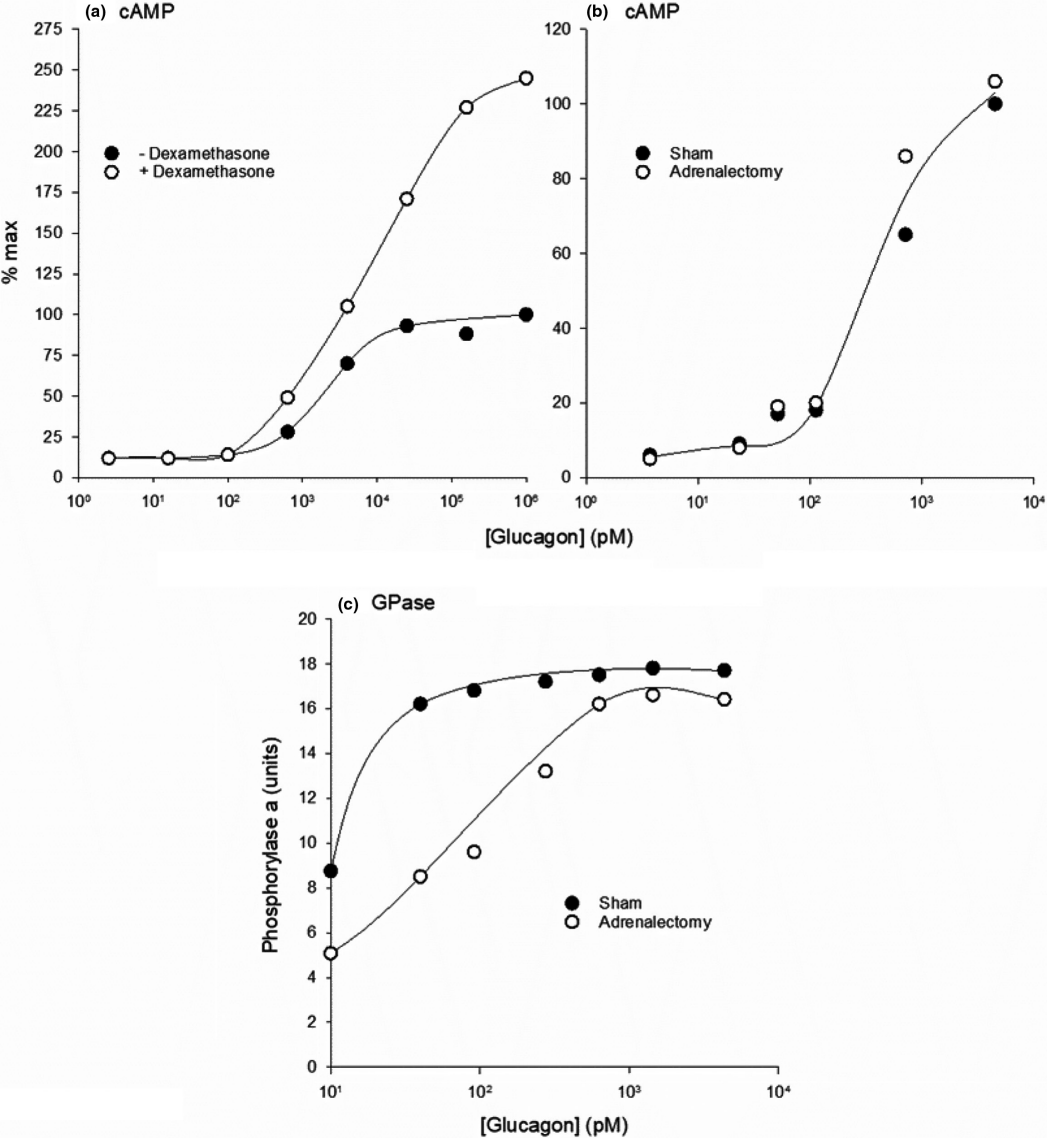
Effects of acute dexamethasone administration ex vivo (a) or prior adrenalectomy (b and c) on glucagon concentration‐dependent increases in cAMP generation (a, b), or phosphorylase a (GPase) activity (c) in rat hepatocytes. Administration of dexamethasone “sensitized” the hepatocytes by increasing the maximal response, but did not alter the TC of 630 (10^2.80^) pM (a). Adrenalectomy in vivo had no effect ex vivo on glucagon‐induced increases in cellular cAMP (b) or PKA activity (not shown). However, adrenalectomy did inhibit the stimulation by glucagon of GPase activity (c) and glucose output (not shown). Note that, in the sham‐operated or untreated group, a concentration of 60 (10^1.78^) pM near‐maximally activated GPase (c), but had minimal effects on tissue cAMP levels (a and b). These results suggest that the “sensitizing” effects of exogenous corticosteroids are apparent ex vivo, but may not be consistently induced by endogenous corticosteroids in vivo (see Figures 7 and 8). As a control, endogenous corticosteroids do seem to contribute to the stimulation of phosphorylase activity by zone 1 and zone 2 concentrations of glucagon in vivo (c). Adapted from references Christoffersen et al. (1984) (a) and Chan et al. (1979) (b and c)

Plasma corticosteroids are elevated in early neonates, exercising adults, starvation, and T1D (Huang et al., 2006; Ogias et al., 2010; Schwartz et al., 1997; Watanabe et al., 1991). In the first two conditions, any influence of elevated corticosteroid levels on the extent of activation of hepatic AC/cAMP by glucagon is obscured by the marked increase in plasma glucagon levels (Figure 2d), well above 100 pM and thus sufficient to consistently and robustly activate AC on its own (Table 4C and D). In starvation and T1D, however, plasma glucagon concentrations do not rise as high; they can either stay in zone 1 (below 60 pM) or increase into the transitional zone 2 (60–100 pM), but very rarely go above 100 pM. Not surprisingly, the influence of glucagon on hepatic AC/cAMP in these conditions is inconsistent, as discussed above. One possible explanation for the inconsistency is that attendant increases in plasma corticosteroid levels have variable effects on the activation of the AC/cAMP pathway and stimulation of glucose output by glucagon at transitional concentrations (Figure 10).

Corticosteroids can either act on their own or interact with glucagon to influence hepatic gluconeogenesis. Glucocorticoids, by interacting with the glucocorticoid receptor and the glucocorticoid response element, activate a number of transcription factors involved in the expressions of PEPCK and G6Pase, ultimately stimulating gluconeogenesis (Jitrapakdee, 2012). They synergize with cAMP to induce the transcriptional activator PGC‐1 and gluconeogenesis (Banzet et al., 2009); PGC‐1 increases expressions of both PEPCK and G6Pase (Yoon et al., 2001). In addition, corticosteroids are often described as having a “permissive” effect on the generation of cAMP by high concentrations of glucagon and by other AC activators such as NaF, isoproterenol, or forskolin (Adigun et al., 2010; Exton, 1979; Exton et al., 1972; Krone et al., 1976; Yoon et al., 2001). The nonselective action has been termed “heterologous sensitization” (Adigun et al., 2010). Liver‐specific knockout of the glucocorticoid receptor suppressed the enhanced expression of PEPCK in both STZ‐diabetic and 48‐h starving normoglycemic mice, and blunted the hyperglycemia in the former and exacerbated the hypoglycemia in the latter (Opherk et al., 2004). Enhancement of glucagon’s AC/cAMP‐mediated effects may also involve glucocorticoid‐induced inhibition of cAMP phosphodiesterase (Manganiello & Vaughn, 1972) and increased expressions of Gαs1 and Gαs2 in both neonates and adults (Kawai & Arinze, 1993).

The permissive or sensitizing effect of corticosteroids on responses to AC‐activating concentrations of glucagon is observed ex vivo but not consistently in vivo. Administration of dexamethasone to cultured rat hepatocytes enhanced the effectiveness of glucagon to increase cAMP levels by displacing the concentration‐effect curve upward (Figure 10a), but without altering its potency (i.e., it did not affect the TC, which in this case was 630 pM). These results suggest that elevations in plasma glucocorticoids would enhance the AC‐activating actions of glucagon in vivo only if plasma glucagon concentrations rise above 100 pM. Consistent with that view, administration of dexamethasone (10 or 50 μg/kg/d, 26d) to metabolically unstressed rats, whose plasma glucagon levels were presumably below 60 pM, had little to no effect on AC activity in rat liver (Slotkin et al., 1996).

It follows that the question of whether the ability of glucagon to inconsistently activate the AC/cAMP pathway at concentrations above 60 pM, in T1D or starvation, can be attributed at least in part to permissive effects of corticosteroids has not been answered definitively. As discussed above, the relationship between plasma glucagon and hepatic AC/cAMP in T1D or starvation is variable (Figures 7 and 8), and not always correlated with changes in plasma corticosteroids. Withdrawal of endogenous corticosteroids by adrenalectomy might be predicted to suppress the concentration‐dependent stimulation of the AC/cAMP pathway by glucagon ex vivo. But that is not borne out by the results shown in Figure 10b; adrenalectomy had no effect on glucagon’s activation of AC or PKA in hepatocytes (Chan et al., 1979; Seitz et al., 1976). As a control (Figure 10c), adrenalectomy did inhibit basal GPase activity and its enhancement in response to glucagon at concentrations between 100 and 7000 pM. It also suppressed the glucagon concentration‐effect curve for glucose output (Chan et al., 1979). In contrast, adrenalectomy slightly *increased* exercise‐induced elevations in hepatic cAMP levels without altering plasma glucagon levels (Sellers et al., 1988). Starvation of rats for 48 h increased serum glucagon (from about 25 to 55 pM), hepatic PEPCK, and tissue cAMP to the same extent in control and adrenalectomized rats, while not markedly affecting serum insulin levels in either group (Seitz et al., 1976).

Glucocorticoids may also influence responses to glucagon that are mediated by the PLC/IP3 pathway, but the evidence is indirect. Adrenalectomy suppressed the stimulation of glucose output or gluconeogenesis in perfused rat livers produced by alpha agonists (Ciprés et al., 1995), which presumably act exclusively via the PLC/IP3 pathway. One implication is that glucocorticoids can enhance glucagon’s effects on glucose mobilization by influencing the PLC/IP3 pathway at concentrations below 60 pM, and both the PLC/IP3 and AC/cAMP pathways at higher concentrations. Establishing the role of endogenous corticosteroids in the control of hepatic glucose mobilization by glucagon in both unstressed and metabolically stressed animals will require further study.

The current, AC/cAMP‐based model of glucagon’s hepatic actions should be revised, and the focus of future research should be redirected accordingly, placing much greater emphasis on the role of the PLC/IP3 pathway.

## 10 CONCLUSIONS AND FUTURE INVESTIGATIONS

**FIGURE 11:**
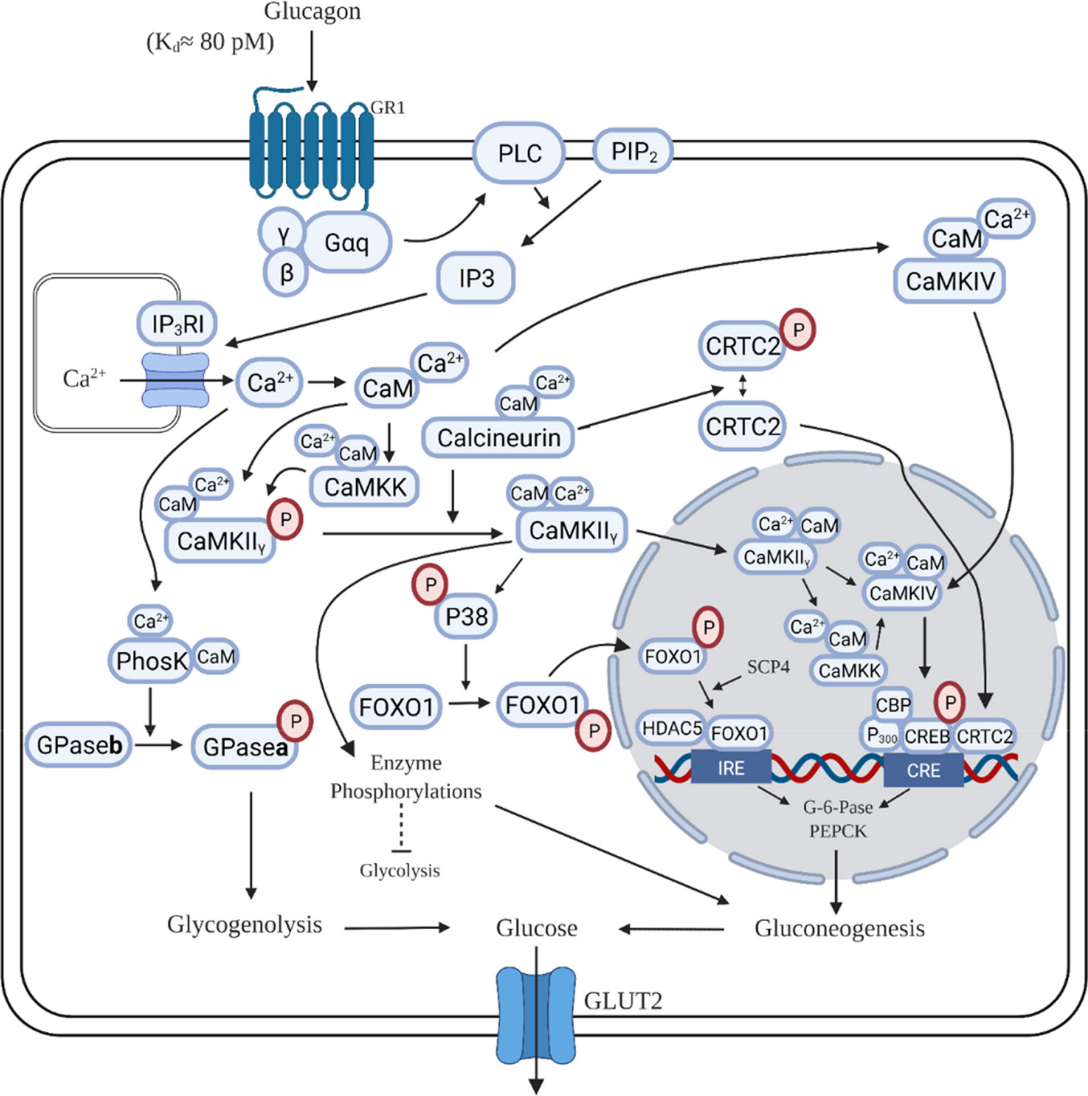
Presumptive model of intracellular events in response to activation of GR1 receptors and phospholipase C (PLC) by glucagon in hepatocytes. Many of the targets of CaMKs depicted here are also targets of PKA, activated by supraphysiological or pharmacological concentrations (see text). The Kd value of 80 pM for the purified receptor is from Andersson et al. (1993). The model for nuclear translocation of CaMKII and its interactions with CaMKK and CaMKIV in the nucleus is from Cohen S et al., 2015 for neurons, and for Fox01 translocation is from Ozcan et al. (2012) for hepatocytes. Possible involvement of AMPK in this model is not firmly established (see text), and therefore is not shown. CaM, calmodulin; CaMKII and IV, calmodulin‐dependent protein kinase II and IV; CBP, CREB‐binding protein; CRE, cyclic adenosine monophosphate response element; CREB, cyclic adenosine monophosphate response element binding protein; CRTC2, CREB transcriptional coactivator 2 (aka TORC2); ER, endoplasmic reticulum; Fox01, forkhead box 01; GR1, glucagon receptor type 1; HDAC5, histone deacetylase 5; IP3, inositol triphosphate; IP3R, inositol triphosphate receptor; IRE, insulin response element; P300, histone acetyltransferase p300; GPasea, glycogen phosphorylase a; GPaseb, glycogen phosphorylase b; PhosK, glycogen phosphorylase kinase; PIP2, phosphoinositol‐bisphosphate; PLC, phospholipase C; SCP4, small C‐terminal domain phosphatase. The figure was constructed (BioRender.com) using information gathered from the following references: GR1(Kd)/ Gαq /PLC/IP3/IP3RI: Andersson et al. (1993), Barucha and Tager (1990), Bill and Vines (2020), Bonnevie‐Nielsen and Tager (1983), Chin & Means, 2000), Goldstein and Hager (2018), Igumenova (2015), Ikezawa et al. (1998), Miura et al. (1992), Newton (2018), Sonne et al. (1978), Takemoto‐Kimura et al. (2017) and Wayman et al. (2011), PhosK/GPase: Brushia and Walsh (1999), Miura et al. (1992) and Vénien‐Bryan et al. (2002), CaMKII: Cohen et al. (2015), Johannessen and Moens (2007) and Takemoto‐Kimura et al. (2017), CaMKIV: Hook and Means (2001), Shaywitz and Greenberg (1999), Skelding and Rostas (2020) and Soderling, 1999), CaMKK: Anderson et al. (2012), Brzozowski and Skelding (2019), Puri (2020), Racioppi and Means (2012) and Skelding and Rostas (2020), Calcineurin/CRTC2: Oh et al. (2013) and Rui (2014), Nuclear translocation of Fox01, CRTC2, CaMKII, and CaMKIV: Cohen et al. (2015), Goldstein and Hager (2018), Ozcan et al. (2012), Skelding and Rostas (2020) and Wayman et al. (2011), Intranuclear: Cohen et al. (2015), Goldstein and Hager (2018), Johannessen and Moens (2007), Müller et al. (2017), Oh et al. (2013), Ozcan et al. (2012), Ravnskjaer et al. (2015), Shaywitz and Greenberg (1999), Soderling, (1999)

If there is one take‐home message of this review, it is this: When it comes to investigating the endocrinology of glucagon, concentration matters. The weight of the evidence indicates that plasma glucagon concentrations are below 60 pM in the absence of metabolic stress or in T2D, and within that range do not activate hepatic AC above basal levels. When they rise above 100 pM in the early neonate or exercising adult, they predictably activate AC to adaptively boost glucose output to meet the elevated systemic glucose demand. In that regard, the adaptive response in exercising adults recapitulates that of the early neonate. Inconsistencies become apparent when glucagon concentrations are between 60 and 100 pM, characteristic of T1D and starvation. Activating AC by high, pharmacological concentrations—above 800 pM—produce responses that are not physiologically relevant and in any case are difficult to interpret because of extensive cross‐talk between the two signal pathways and because the intracellular targets of the two pathways overlap considerably (Figure 11).

“Uncertainty” in the title of this review refers to a number of important unanswered questions, including: (1) What are the respective roles of the PLC/IP3 and AC/cAMP pathways in the regulation of glucose output by glucagon in the absence and presence of metabolic stress? (2) Exactly how low is the glucagon concentration in hepatic portal plasma, and how, precisely, does its concentration vary in response to various metabolically stressful conditions? (3) At what concentrations and to what extent does glucagon activate the hepatic PLC/IP3 pathway in the early neonate? (4) Which intracellular downstream targets are common, and which are unique, to the AC/cAMP and PLC/IP3 pathways? (5) As a target of the PLC/IP3 pathway, what is the role of AMPK in glucagon’s regulation of hepatic glucose mobilization in vivo? and (6) How do elevations in circulating glucocorticoids influence the regulation of hepatic glucose mobilization by glucagon under normal, metabolically‐unstressed conditions, as well as in T1D or starvation?

Uncertainties remain, largely because focused experiments specifically designed to directly address the central questions posed in this review are rare. New studies blending classical pharmacological and modern molecular or gene manipulation approaches stand a good chance of filling at least some of the information gaps. For example, dose‐response curves depicted in Figure 4 could be repeated, but employing techniques and experimental approaches that are extensions and refinements of those used by Sutherland and coworkers a half‐century ago. The isolated perfused rat liver preparation that they and others have utilized can be replaced with an isolated mouse liver preparation, adapted for perfusion with glucose and fatty acids (Ferrigno et al., 2013; Harney & Rodgers, 2008), to allow the application of both pharmacological and gene manipulation techniques in the same study. If the portion of the glucagon‐glucose mobilization curve that is below 60 pM shown in Figure 4 is displaced rightward by pretreatment with, for example, the PKA blocker H89 (Lochner & Moolman, 2006), by liver‐specific PKA inhibition as discussed above (Willis et al., 2011), or by knocking out appropriate Gαs subtypes, then the results would support the alternative hypothesis that activation of the AC/cAMP pathway does contribute to the mediation of the glucose‐mobilizing effects of glucagon at physiological concentrations in vivo. If, however, that portion of the curve is only affected by interventions such as pretreatment with the PLC antagonist U73122 or a selective Gαq inhibitor such as GP2A or YM‐19 (Zhang et al., 2020), or by knocking out the appropriate Gαq protein, then the results would support the central hypothesis proposed here, that glucagon regulates hepatic glucose mobilization exclusively via the PLC/IP3/CaM pathway at physiological concentrations. A glucagon‐AC/cAMP dose‐response curve like the one depicted in Figure 4 would serve as a control for both the effectiveness of anti‐AC/cAMP interventions and the specificity of PLC or Gαq inhibitors. Experiments of this kind would help to satisfy an unmet need in the characterization of glucagon’s true mechanism of action.

The longstanding cAMP bias should be seriously reexamined. It may have persisted this long because of the widespread reluctance to acknowledge the substantial gap between hepatic portal glucagon concentrations and the minimal concentration required to activate AC, along with an underappreciation of the efficacy and complexity of the GR1/PI3/IP3/Ca^2+^/CaM pathway (Figure 11). Too often in experimental reports or review articles, the PLC/IP3 pathway has been acknowledged only in passing or even ignored altogether (e.g.,Christophe, 1995; Goldstein & Hager, 2018; Jiang & Zhang, 2003; Mauger et al., 1989; Rix et al., 2019; Schwartz et al., 1997; Wewer Albrechtsen et al., 2016), although there are welcome exceptions (e.g Müller et al., 2017; Perry et al., 2020). In the future, more attention should be paid to the detailed characterization of the hepatic GR1 receptor complex and downstream events, as well as to the interactions between the PLC/IP3 and AC/cAMP pathways, particularly at physiological and pathophysiologically relevant hormone concentrations. The administration of very high, pharmacological concentrations ex vivo, often at the extreme concentration of 100,000 pM, yields information that is applicable to the cellular consequences of a maximally activated AC/cAMP pathway, but generates intracellular responses of questionable relevance to the physiological or pathophysiological effects of the hormone on the liver in vivo. Given the increasing appreciation of glucagon’s central role in the etiology of diabetes (Burcelin et al., 1996; Johnson et al., 1982; Lee et al., 2012; Patil et al., 2020; Unger & Cherrington, 2012; Wewer Albrechtsen et al., 2019), resolving persistent uncertainties and establishing its true mechanism of action in health and disease are now more urgent than ever.^1^

## FUNDING INFORMATION

The author has received no external funding in support of this manuscript.

## CONFLICT OF INTEREST

The author declares no conflict of interest.

## Data Availability

No studies on experimental animals or humans were carried out for this manuscript.

## Footnotes

^1^
For reviews on this and related topics, see Adeva‐Andani et al. (2019), Agius (2007), Almahariq et al. (2014), Altarejos and Montminy (2011), Barthel and Schmoll (2003), Bill and Vines (2020), Blunsom and Cockroft (1865), Brushia and Walsh (1999), Burcelin et al. (1996), Campbell and Newgard (2021), Carling et al. (2011), Chin and Means (2000), Christophe (1995, 1996), Cohen (1989), Cohen et al. (2015), D’Alessio (2011), Dunning and Gerich (2007), Engin et al. (2017), Exton (1979, 1986, 1987, 1993), Exton et al. (1981), Foretz et al. (2005), Fullerton (2016), Girard (1986, 1990), Güemes et al. (2016), Han et al. (2016), Hasenour et al. (2013), Hook and Means (2001), Jacquel et al. (1983, 2014), Janah et al. (2020), Jiang and Zhang (2003), Jitrapakdee (2012), Kaira et al. (2021), Klein and Malviya (2008), Kraus‐Friedmann (1984), Ktorza et al. (1985), Lin and Accili (2011), Mayo et al. (2003), Mayor and Cuezva (1983, 2014), Miller and Birnbaum (2016), Moore et al. (1998), Mortimore and Pösö (1987), Müller et al. (2017), Newton (2018), Nimmo and Cohen (1977), Nishizuka (1989), Okar et al. (2004), Ong and Ambudkar (2020), Patil et al. (2020), Pilkis and Ghranner (1992), Racioppi and Means (2012), Raju and Cryer (2005), Ravnskjaer et al. (2015), Rider et al. (2004), Rix et al. (2019), Rodgers (2012, 2021), Rui (2014), Servillo et al. (2002), Sharabi et al. (2019), Soderling (1999), Sugden et al. (1993), Taborsky (2010), Unger (1985), Unger and Cherrington (2012), Unterman (2018), Viollet et al. (2009), Wendt and Eliasson (2020), Wewer Albrechtsen, et al. (2016), Wicks (1971), Williamson et al. (1987), Winder (1985), Wu‐Zhang and Newton (2013), Xing et al. (2018), Yabaluri and Bashyam (2010), Yang and Yang (2016) and Zhang, et al. (2019).
